# Deep DNA and protein level feature integration for robust clinical variant interpretation using probabilistic gradient boosting

**DOI:** 10.3389/fdgth.2026.1845955

**Published:** 2026-08-26

**Authors:** V. Karthik, S. Prejesh, Sumedh Deepak Kudale, Chandra Umakanthan OmKumar

**Affiliations:** School of Computer Science and Engineering, Vellore Institute of Technology, Chennai, India

**Keywords:** clinical genomics, ClinVar, probabilistic gradient boosting, protein structure, variant interpretation, variant pathogenicity

## Abstract

A major challenge in clinical genomics is to classify genetic variations correctly, since it directly affects disease diagnosis and personal care. The existing methods tend to be based on the combination of different factors, such as protein structure, population frequencies, phenotypic annotations, and sequence conservation. Nevertheless, these methods often cannot be used to achieve the necessary interpretability, quantify uncertainty, and address rare cases. This paper presents a probabilistic gradient boosting model on variant pathogenicity prediction. The suggested framework applies biological characteristics at both level of DNA and protein levels while also scaling the level of uncertainty in clinical decision making. Our machine learning aims to solve the issues of variant interpretation by managing the features and through probability-based pathogenicity prediction. The framework formulation is aimed at generalizing over various datasets and minimizing overfitting. At the same time, it can ensure reasonable performance to facilitate clinical experiments. The model has also been tested on three standard datasets and demonstrated to be more predictive of the pathogenic effect of variants, in comparison with a variety of existing tools. The probabilistic gradient boosting model proposed had ROC AUC values of 0.9293, 0.9610, and 0.9646 on ClinVar variants, GRCh37, and GRCh38 human genome respectively. Furthermore, the dataset was ensured to include both exonic and intronic variants, and Variants of Uncertain Significance were also taken into consideration for Performance Testing. Through this it also aims to provide better clinical significance which will lead to a good interpretable tool for priority of variants for a large variety of disease conditions.

## 1 Introduction

Genetic variation is fundamental to defining the topography of human diversity and clinical pathology (1). While most variants are classified as neutral, a specific proportion disrupts regulatory mechanisms, potentially destabilizing proteins or disrupting gene activity, which manifests as pathogenic effects (2). A major challenge in medical genetics and precision medicine delivery is distinguishing these harmful pathogenic variants from harmless polymorphisms. This accurate variant effect prediction is of paramount importance and heavily impacts early diagnosis, treatment planning and targeted therapeutics for many disease areas. The task is complex and multifaceted, and is subject to sequence conservation, genomic context, structure and epigenetic control. Traditionally, variant interpretation methods have been based on the consensus of experts and manually curated databases. However, these resources are informative, but do not cover the scale of novel variants produced by the high-throughput sequencing technologies. Other limitations include the high percentage of variants of uncertain significance (VUS), highlighting the need for new and innovative approaches that are not based on rules or heuristics [3]. Concurrently, computational approaches have increasingly been embraced as a routine tool for prediction of variant pathogenicity. The main drivers of this shift are recent breakthroughs in machine-learning and the availability of abundant and rich genomic datasets [4]. Current machine learning methods combine diverse features such as evolutionary conservation, protein structure and functional annotations. Recent tools such as deep learning and ensemble models have emerged and have become more accurate than the former ones [5]. Most conventional approaches, however, lead to deterministic classifications which are unable to properly capture and express the uncertainty in the predictions. In the clinical world, the uncertainty quantification is as important as the accuracy: not only clinicians need a classification label but they also need a calibrated measure of predictive confidence. These confidence metrics are essential for computational predictions to be part of clinical workflows. Quality and availability of training data are further challenges. The number of pathogenic examples in variant datasets is often not significantly large compared to benign examples and there are inconsistencies for the clinical interpretation changing over time [6]. This imbalance can cause models to be overfitting, resulting in them having poor generalization capabilities to novel or rare variants. In addition, the use of high-dimensional features such as embedding of protein language models has been shown to improve performance, but it suffers from poor interpretability, which makes it difficult to be used in the clinic. We need methods that can accurately quantify and communicate predictive uncertainty that are interpretable, and that make this uncertainty explicit. To try and fill this gap, we propose a pathogenicity classification framework inspired by probabilistic gradient boosting. Gradient boosting was chosen because it has proven to be successful on genomic tasks and its ability to model non-linear relationships between complex features in the data without sacrificing feature interpretability. We extend this architecture by adding a probabilistic part to the model. The method yields a pathogenicity prediction as well as a quantified uncertainty. Our approach generates both metrics, which provide highly informative, clinically actionable outputs that are not based simply on binary classification, but on confidence intervals or probability distributions.

While the use of gradient boosting machines and other tree ensembles to genomic prediction is established, therefore our goal is to adjust a probabilistic boosting formulation for clinical variant interpretation. The novelty of our approach is that it provides high-level predictive performance in conjunction with explicit uncertainty quantification. The output of our probabilistic model captures the inherent ambiguity of variant information and is optimized for high-stakes clinical decisions (as opposed to the traditional deterministic classifiers). We exploit a heteroscedastic likelihood to model the mean pathogenicity scores along with the predictive variance, which is dependent on the input, allowing the development of calibrated probability outputs and uncertainty-aware decision rules in a standard boosting back-bone. Further, there is also a considerable degree of flexibility in the framework which can be used with different types of variants and multi-domain datasets without any special tuning. A focused set of variants from the evaluation data set consisted of missense, intronic, frameshift, nonsense and UTR variants. Additionally, the model was tested on specimens of uncertain significance with good model performance. Thus, this work provides a computational tool that is able to connect variant identification with clinical interpretations. The novelty of this study is in the systematic combination of multi-layered DNA-level and protein-level features with clinically-oriented uncertainty quantification, implemented around the framework of gradient boosting.

The proposed framework combines uncertainty-aware prediction with probabilistic gradient boosting, thereby enabling clinical variant interpretation and overcoming limits of conventional pathogenicity prediction techniques, which yield deterministic pathogenicity predictions. The model is based on a combination of DNA level and protein level descriptors such as sequence composition, structural stability, evolutionary conservation, and physicochemical properties, to provide pathogenicity scores and calibrated confidence estimates. The framework also includes a feature attribution analysis, an ablation study, and a hypothesis-generation module that investigates the sequence changes, which are biologically plausible, corresponding to reduced predicted pathogenicity. The combination of these components leads to a more understandable and uncertainty-aware approach for genomic variant prioritization.

Present computational methods for predicting the pathogenicity of variants have a number of drawbacks, such as inadequate uncertainty quantification, limited interpretability, limited applicability to rare variants, and variability in performance on different and heterogeneous genomic datasets. The present research aims to address these limitations by developing a probabilistic machine learning framework capable of generating accurate pathogenicity predictions together with calibrated confidence estimates while integrating biologically meaningful DNA- and protein-level features.

### 1.1 Contributions

- To build a probabilistic gradient boosting model for variant pathogenicity classification that offers high predictive accuracy and also improves interpretability.
- Probabilistic approach considers the ambiguity of variant pathogenicity instead of relying solely on binary labels.
- The feature engineering process incorporates a range of diverse biological features including protein structure and DNA sequences.
- To propose some biologically possible mutations that could reduce the predicted pathogenicity.

This study is organized as follows: Section 2 deliberates the current research on machine learning-based tools for predicting pathogenicity of variants. The proposed framework using probabilistic gradient boosting model is described in Section 3 of the paper. Results and discussion are explained in Section 4. The conclusion and future scope are discussed in Section 5.

## 2 Related work

There has been a transformation toward using specialized machine learning architectures for different classes of variants in the field. Schuetz et al. [7] have developed a tree-based machine learning method for the classification of the clinical relevance of copy number variants (CNVs), called CNVoyant. In order to capture the variables affecting CNV impact, the model features a set of features that includes gene density, centromere distance and GC content. While CNVoyant performs at an ROC AUC of 0.858, the use of static training sets means that there is the potential for bias in application to new patient cohorts. In the same way, Liu et al. [8] developed a supervised machine learning protocol (NeuroCNVscore) to focus on the pathogenicity of CNVs linked to neurodevelopmental disorders (NDDs). This method aims to simulate CNV effects on neurological phenotypes by integrating enhancers and patterns of gene expression in the brain. Although the model is effective in a specific context, its overall clinical usefulness is limited due to the need for optimal quality tissue-specific annotations. They also compared their performance to other databases of variants, including ClinVar, to assess clinical relevance. This neuro-centric emphasis, together with the apparent lack of generalizability to non-neural pathologies and the reliance on brain annotation datasets illustrates a fundamental balance of specialization in tissue versus generalizability. In a different area of interest, Sharo et al. [9] designed StrVCTVRE, a structural variant (SV) pathogenicity pipeline that used properties of the structural variations such as the overlap of the exon with the gene, potential loss-of-function impact, and conservation metrics to feed a random forest classification system. StrVCTVRE has competitive performance in deletions, duplications and various types of rearrangements, but is trained using curated pathogenic (clinically significant) and benign (common) SV examples from ClinVar. This creates a significant limitation because of the inherent heterogeneity of SVs and imprecision in breakpoints, making feature extraction more complicated, and limiting robustness to complex SV rearrangements.

Haque et al. [10] used somatic cancer mutation signals to infer the germline pathogenicity. The authors showed that somatic recurrence can provide clues to germline pathogenicity and identify key germline mutations at functionally relevant residues by training on large somatic mutation cohorts and mapping these signals to variants that are known to be pathogenic to the germline in ClinVar. The distinct evolutionary pressures that are present in somatic versus germline environments may lead to confounding signals; here the model has an ROC AUC of 0.821.

In recent years, advanced representation learning through protein language models (PLMs) has been developed, such as VariPred [11]. When compared to ClinVar standard pathogenic and benign datasets, the PLM features enhance classification accuracy, especially for the variants that do not have resolved structural information. But another drawback of these PLM approaches is that the latent embeddings are a black box and would require post-hoc attribution methods for clinical use. Indeed, the authors concede that these embeddings are inherently unexplainable, and that, without the help of additional attribution mechanisms, these embeddings cannot be directly interpreted in a clinical context.

In order to prioritize pathogenic CNVs, Hertzberg et al. [12] devised a framework called TADA that relies on functional data in the form of gene constraint scores and regulatory overlaps to prioritize genes for cohort-based gene discovery. Using machine learning, the TADA framework combines and ranks these features, outperforming the rule-based approaches on curated CNV datasets. However, a few limitations of TADA warrant further attention: the absence of mosaic CNVs, which are common in complex clinical samples, and the absence of a breakpoint imprecision in the detection of CNVs, which can often compromise the accuracy of the ranking.

To prioritize the genes affected by structural variants, Xu et al. [13] generated a new and interpretable method called PhenoSV, which combined molecular effect features with Human Phenotype Ontology (HPO) based phenotype similarity scores. In instances where there was a large amount of phenotyping data, evaluated on patient SV datasets with curated clinical SV databases such as ClinVar, phenoSV improved the AUC to 0.91. One major challenge with this approach is that it requires a lot of granular phenotypic data – if clinical information is noisy or not complete, it will have a significant impact on how well this works in the real world.

Kumaran and Devarajan [14] created a random forest pipeline, named eyeVarP, which was specifically optimized for the detection of eye diseases using eye-related annotations for the genes. By using clinical variant references, the study shows that candidate identification for eye disease datasets can be significantly enhanced, but this strict specialization reduces the amount of cross domain reuse, and requires the curation of clinical variants, which may not exist for less well-characterized diseases.

To directly capture the uncertainty and inherent learning biases of Mendelian reference data, Requena et al. [15] presented a method named CNVscore to approximate pathogenicity. This approach improves the clinical interpretability by combining machine learning scores with quantitative confidence intervals. While CNVscore's uncertainty estimates give better clinical interpretability than existing, static curated CNV resources. However, there is a need for more independent validation and calibration of the technique in a variety of clinical cohorts before it can be widely used.

Pei and Grishin [16] developed a multifunctional tool named DBSAV that combines both clinical and computational data into a unified platform that functions as a single-amino-acid variant curation database and a deleterious predictor. DBSAV provides a comprehensive database and a basic prediction of single-residue effects, using open source variant databases like ClinVar and experimental data. A key drawback of DBSAV is that unlike macro-variant tools, it can only model individual residues, making it a smaller platform and not a full source for SV/CNV interpretation.

A different study showed that AlphaFold2-predicted descriptors when added to a random forest architecture significantly boost missense pathogenicity classification [17]. With the ClinVar benchmark dataset, the study demonstrates that the AlphaFold2-based descriptors are highly discriminative between pathogenic and benign substitutions with an AUROC of 0.793. However, the authors also note that the uncertainty that goes with the uncertainty of the structural confidence of the predictions, particularly in intrinsically disordered or flexible protein regions, is also a big limitation for interpretation.

One such instance of gene specific modelling is illustrated by Lin et al. [18] who focused on risk stratification of BRCA1 variants of uncertain significance (VUS) using targeted clinical datasets and supervised learning. They have developed a more effective risk stratification for BRCA1 VUS than generic predictors and this aids gene-centric interpretation strategies. The downside to this high accuracy is that such optimized and specialized parameters are not straightforward to apply to other cancer-susceptibility genes, unless the high-quality curated data exists as with the BRCA1 data.

Joudaki et al. [19] developed a LightGBM-based architecture, FexSplice, that predicts the splicing effects of single-nucleotide variants (SNVs) based on local context and splice motifs. When trained and tested on curated splicing assay data, FexSplice achieves excellent performance on this class of highly constrained splice variants. A limitation of FexSplice is that it only works for G-transitions at exon boundaries, which means additional models are needed to achieve broad coverage over a larger set of splicing variants.

Won et al. [20] created an evolutionary constraint model, 3Cnet, based on a deep-learning model that simultaneously uses evolutionary constraint signals and additional prediction tasks to accurately predict pathogenicity in a wide range of genes. This neural network architecture is able to learn shared representations, which improves the robustness and performance of the network across ClinVar and other benchmark variant datasets by joint training on interrelated tasks. While this multitasking approach helps stabilize the model, careful balancing of tasks is required to prevent negative transfer and it is very dependent on the quality of labels for the auxiliary tasks.

Moyon et al. [21] proposed a framework named FINSURF that combines evolutionary conservation, regulatory annotations, and epigenomic signals, to pinpoint variants of high pathogenic potential. In their study they showed excellent performance in supervised learning to distinguish pathogenic and benign non-coding variants. The model is nevertheless useful and has several limitations that inhibit its generalizability to the rest of the world, however: it has difficulty modeling tissue-specific regulatory dynamics, and it fails to incorporate functional validation data.

Ciesielski et al. [22] hypothesized that existing binary classification schemes fail to account for the effects of environmental factors, phenotypic variation and sex. By analyzing more than 5,000 ClinVar variants from the BioMe and UK Biobank cohorts, they were able to estimate an average penetrance of 7%. Their sex-stratified alleleratio analysis of the findings highlights the importance of context-sensitive predictive models as variants traditionally thought to be benign can show conditional lethality.

To clarify the ambiguities of the gene variants associated with hypophosphatasia, Farman et al. [23] founded the Global ALPL Gene Variant Classification Project. They used systematic application of the ACMG/AMP guidelines, phenotypic scoring, functional assessments and CRISPR-based assays to classify variants into pathogenic and benign groups within their curated framework. This integrated, ClinVar linked database is a modern model of variant classification in rare diseases and will provide a significant methodological model for future gene-specific curation efforts.

Bergquist et al. [24] critically assessed the performance of VARITY, ESM1b and AlphaMissense when using ClinVar datasets and normalized their raw output to the ACMG/AMP clinical thresholds. They applied a Bayesian approach to transform the continuous computations into likelihood ratios, representing different degrees of clinical evidence, and found that VARITY consistently gave strong evidence h when it was either benign or pathogenic. This validation showed the powerful applicability of these methods and underscored the need for systematic recalibration in the face of out-of-the-box developer defaults.

The Critical Assessment of Genome Interpretation (CAGI) Consortium [25] assessed 738 submissions for 50 separate challenges, and concluded that although the relative performance of the best results is generally good, the absolute effect estimates differ greatly. Predictions on ClinVar benchmarks were within the range of 0.85-0.92, with ensemble methods like REVEL successfully creating Supporting or Strong ACMG/AMP evidence; however, there is still a significant amount of work to be done to close the gap between such models and variant interpretation workflows.

Lin et al. [26] published VIPdb version 2, which is a comprehensive database of 407 variant impact predictors over the last 30 years. These tools range from supervised to unsupervised methods and more and more focus on insertions and deletions (indels), splicing and regulatory variants. VIPdb integrates metadata from ClinVar, ACMG/AMP guidelines and CAGI benchmarks, creating a well-structured resource to navigate the growing complexity of computational predictors.

We present PROBC (28) which is a generative probabilistic approach to the decomposition of Hi‐C and Micro‐C interaction patterns into contributions from histone modifications and transcription factor binding profiles based on convex likelihood optimization. Chromatin marks that directly predict chromatin contacts—including H3K27ac, H3K9me3, H3K4me3, H3K4me1, and CTCF—show that these interactions are generalizable across cell types and species in PROBC. Unlike PROBC, our framework deals with the classification of the pathogenicity of variants in a probabilistic fashion based on genomic and protein features that are used as inputs, and the outputs are calibrated for clinical interpretation. Both methods highlight the importance of probabilistic modelling for the genomics: PROBC as a tool of genome architecture, and our approach as a tool of variant pathogenicity under uncertainty.

Eralp and Sefer [29] combined Hi-C with splicing QTL (sQTL) mapping in 8 cancer tissues, finding that when compared to matched controls, sQTLs and their target genes are more likely to be located in the same TADs and they are more likely to have more interactions with chromatin. They also find that tissue-specific sQTLs tend to cluster in tissue-specific Frequently Interacting Regions (FIREs), and that there is a dose-independent positive correlation between tissue specificity of the sQTL and the degree of enrichment in tissue-specific Frequently Interacting Regions (FIREs) even when controlling for eQTLs and genomic distance. The results corroborate the paradigm that pathogenic and regulatory variants are likely to be functionally constrained within the structurally constrained 3D environment of chromatin that regulates the phenotypic impact of sequence changes. Their analysis is complementary to the demonstration that there is a statistical association between 3D genome architecture and splicing regulation in cancer tissues, which we use to build a supervised classifier that uses both DNA- and protein-based biomarkers to assign a calibrated pathogenicity score to each variant. We believe that we can usefully extend our framework with features reflecting this 3D context, e.g. by applying PROBC-like chromatin mark representations or by applying sQTL–Hi-C interaction signals.

Pathogenic Missense scores are generated at a proteome-wide scale using AlphaMissense [30] which combines protein language models with AlphaFold2 structural context. Similarly, EVE [31] employs multiple sequence alignments to build unsupervised deep generative models to estimate the probabilities of variants based on evolutionary constraints without the need for clinical labels. Moreover, the method developed by PrimateAI [32] and its descendants uses deep learning to study the variation between the two populations of primates and humans to classify deleterious from tolerated missense variants throughout the genome. To add perspective to our results based on probabilistic gradient boosting across a range of reference datasets, we employ additional baselines in our experiments: AlphaMissense, EVE, and PrimateAI (GRCh37) as reference datasets to do a point-by-point comparison with our feature-based approach.

In all of these studies, there's a consistent picture that incorporating multiple classes of information (such as sequence conservation, structural metrics, phenotype annotations, and protein embeddings) is much more effective for variant pathogenicity classification than using any single class of information by itself. We suggest a probabilistic gradient boosting perspective in line with this consensus, whereby these multi-omic features are combined into a structure that naturally generates calibrated uncertainty estimates. Adopting probabilistic outputs allows predictive uncertainty to be easily quantified, which is an important obstacle to clinical application. In our model, the feature weighting inherent in the gradient boosting approach, combined with explicit posterior probability estimation, addresses some of the challenges of limited variation, breakpoints with imprecise positions, and of the latent embedding in case of structural variation. These attributes provide thus a promising, interpretable, and clinically actionable approach to strong pathogenicity classification.

## 3 Proposed method

**Figure 1:**
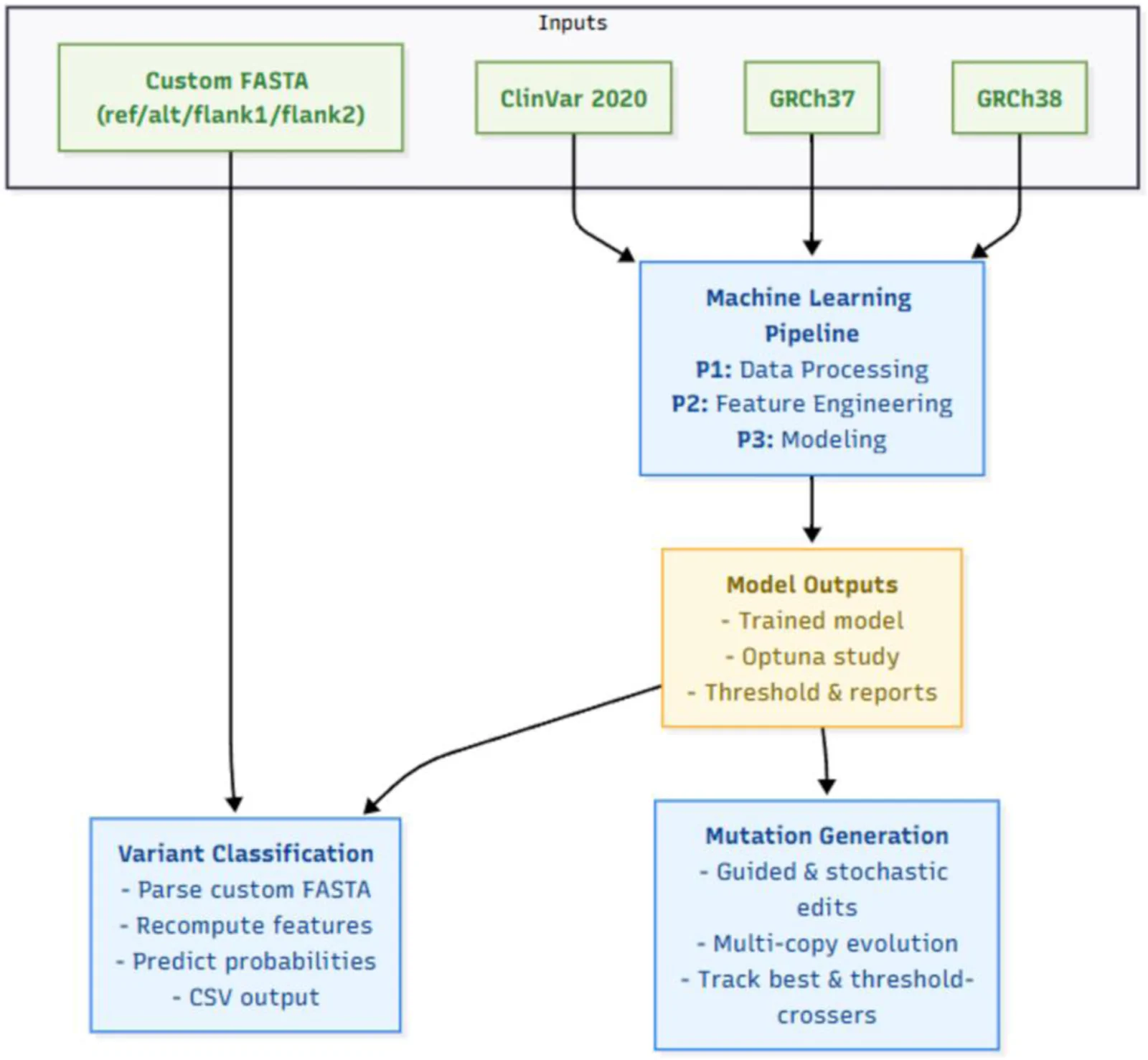
Workflow of proposed pathogenic prediction framework.

Our machine-learning framework to predict pathogenicity is a combination of probabilistic modelling, feature extraction and common source of collected data into one pipeline. The computational code commences with an integration procedure of selected inputs of standardized benchmarks, e.g., ClinVar and human reference genomes, and user-specified FASTA sequences. These inputs undergo automated parsing, sifting and contextualization. These data are then converted into DNA- and protein-based representations and they are intended to encode the key structural and functional properties of the variants. A probabilistic gradient boosting classifier then uses the features to make uncertainty estimates and pathogenic predictions. The final part of the workflow also includes modules for inference on new sequences (FASTA input) and a mutation generation module that proposes some candidate edits to minimize the predicted pathogenicity. The overall workflow of the proposed pathogenicity prediction framework is depicted in Figure 1.

To reduce overlap between the training and evaluation sets, transcript identifiers and variant annotations were also examined, in addition to deduplication occurring at the coordinate level. Coordinate-level deduplication was done in addition to examining transcript identifiers and variant annotations to reduce the overlap between the training and evaluation sets. Alternative transcript variants of the same genomic event were left in the same partition. In addition, variants from several genome builds were analyzed separately, and variants were never moved between experimental data sets. These precautions have been taken to limit information leakage and avoid over-estimating performance.

### 3.1 Data acquisition and preprocessing

Variant data are collected from three sources: ClinVar 2020, and the GRCh37 and GRCh38 genome builds. Each dataset is processed separately, and training and evaluation is carried out on the individual assemblies to assess the generalizability across genomic references.

To avoid information leakage due to the same variant appearing multiple times across data sources, we applied a two-stage deduplication protocol. First, within each dataset (ClinVar 2020, GRCh37, GRCh38), variants were collapsed by chromosomes, genomic coordinate, reference allele, and alternate allele, retaining a single consensus label when multiple records shared identical genomic attributes. Second, when constructing separate assemblies for model development, we ensured that no exact duplicate variant (same chromosome, position, REF, ALT) was shared between training and test folds within a dataset, and that GRCh37- and GRCh38-mapped instances of the same underlying variant were only ever evaluated within their respective build-specific experiments. This design ensures that there are very high levels of separation between train and evaluation splits as well as eliminate the optimistic performance estimates through the duplication across datasets.

Contextual data about all the variants are pulled to unravel regulatory and functional indicators. Fixed-size flanking window of 500 base pairs (±) is read along with each mutation so that the local sequence signals as well as the proximal sequence signals would be provided to be used in downstream feature construction.

### 3.2 Feature engineering

To properly classify the variants, one must have that the raw genomic sequences have to be converted into informative numerical features that can be properly represented by the machine learning model. In order to accomplish this, the proposed framework extracts both DNA and protein feature. The multi-level data is then synthesized into the composite representation that shows biochemical, structural and evolutionary properties of each variant. This helps to make more correct decisions regarding the pathogenicity.

#### 3.2.1 DNA-level features

The difference between the reference and alternative alleles are first measured by using sequence alignment scores. Global (Needleman–Wunsch) and local (Smith–Waterman) alignments are both employed from which similarity and coverage scores are calculated. A generalized similarity index is given below as (Equation 1)

$$\mathrm{Sim}=\frac{M}{M+S+G}$$ (1)

where *M* denotes the number of matches, S the substitutions, and G the gaps introduced during alignment.

The nucleotide composition is further described by GC content, which is highly correlated with gene regulation and replication timing. It is calculated according to (Equation 2)

$$GC\text{\%}=\frac{G+C}{A+T+G+C}\times 100$$ (2)

where A,T,G,C represent the counts of the respective nucleotides in the flanking window.

The nucleotide distribution's Shannon entropy is used to quantify sequence complexity. This metric highlight whether a variant is in regions that are highly conserved or repetitive and is represented in (Equation 3)

$$H=-\overset{n}{\underset{i=1}{\sum }}\;p_{i}\log_{2}\;p_{i}$$ (3)

where $p_{i}$ is the frequency of nucleotide *i*.

The Hairpin or stem-loop structure is predicted, and nearest-neighbor parameters are used to estimate thermodynamic stability. Therefore, structural stability is also considered. The change of free energy due to stacking interactions is given by (Equation 4)

$$\Delta G=\sum_{i=1}^{k}\Delta G_{nn}(b_{i},b_{i+1})$$ (4)

where $\Delta G_{nn}$ is the free energy contribution of neighboring bases $b_{i}\;\;\mathrm{and}\;\;b_{i+1}$.

Lastly, the traits unique to the mutation are included. These include codon changes such as premature stop codons, frameshift indicators for indels, and transition/transversion indicators. Direct disruption of the transcription factor binding sequences also indicates motif disruption.

#### 3.2.2 Protein-level features

Amino acid–level features are isolated to evaluate the specific biochemical and structural disruptions caused when variants modify coding sequences. The translation process identifies potential open reading frames (ORFs) by leveraging codon usage patterns and Kozak sequence motifs. To maintain high experimental precision, ORFs associated with low-confidence metrics are systematically excluded from the analysis.

Subsequent measurement of the physicochemical properties of mutant proteins is done. Calculation of Molecular weight is done directly from amino acid composition whereas the isoelectric point (pI) is estimated as the pH at which the protein has zero net charge. Net charge at physiological pH is represented as per (Equation 5)

$$Q=\sum_{\;j=1}^{m}\left(q_{j}\cdot n_{j}\right)$$ (5)

where $q_{j}$ is the charge of amino acid type *j* at pH 7.4 and $n_{j}$ is its count.

Hydrophobicity changes are measured by the Kyte–Doolittle hydropathy index. Equation 6 expresses the shift caused by mutation

$$\Delta H=H_{\mathrm{mut}}-H_{\mathrm{ref}}$$ (6)

where $H_{\mathrm{ref}}$ and $H_{\mathrm{mut}}$ are the hydropathy scores of the reference and mutant residues, respectively.

Structural descriptors are further obtained by predicting secondary structure propensities and tracking the proline content which is known to influence folding. In addition, substitution likelihoods are quantified using BLOSUM substitution matrices. For a given mutation the calculated substitution score is shown in (Equation 7)

$$S_{\mathrm{sub}}=M(a_{\mathrm{ref}},a_{\mathrm{mut}})$$ (7)

where *M* is the matrix value comparing reference amino acid $a_{\mathrm{ref}}$ with mutant $a_{\mathrm{mut}}$.

Conservation across species is also considered, in that highly conserved residues are more likely to be functionally important. Conservation scores are incorporated as continuous features in order to provide evolutionary context.

#### 3.2.3 Composite representation

All DNA- and protein-level features computed in the previous two steps are concatenated into a single vector representation as shown below in (Equation 8):

$$F=[\;f_{1},f_{2},f_{3},\ldots ,f_{m}]$$ (8)

where $f_{i}$ denotes an individual feature and *m* the total dimensionality. This is the input to the probabilistic gradient boosting classifier hence making sure that the sequence level and the protein level information are used to achieve the ultimate classification.

### 3.3 Machine learning pipeline

**Figure 2:**
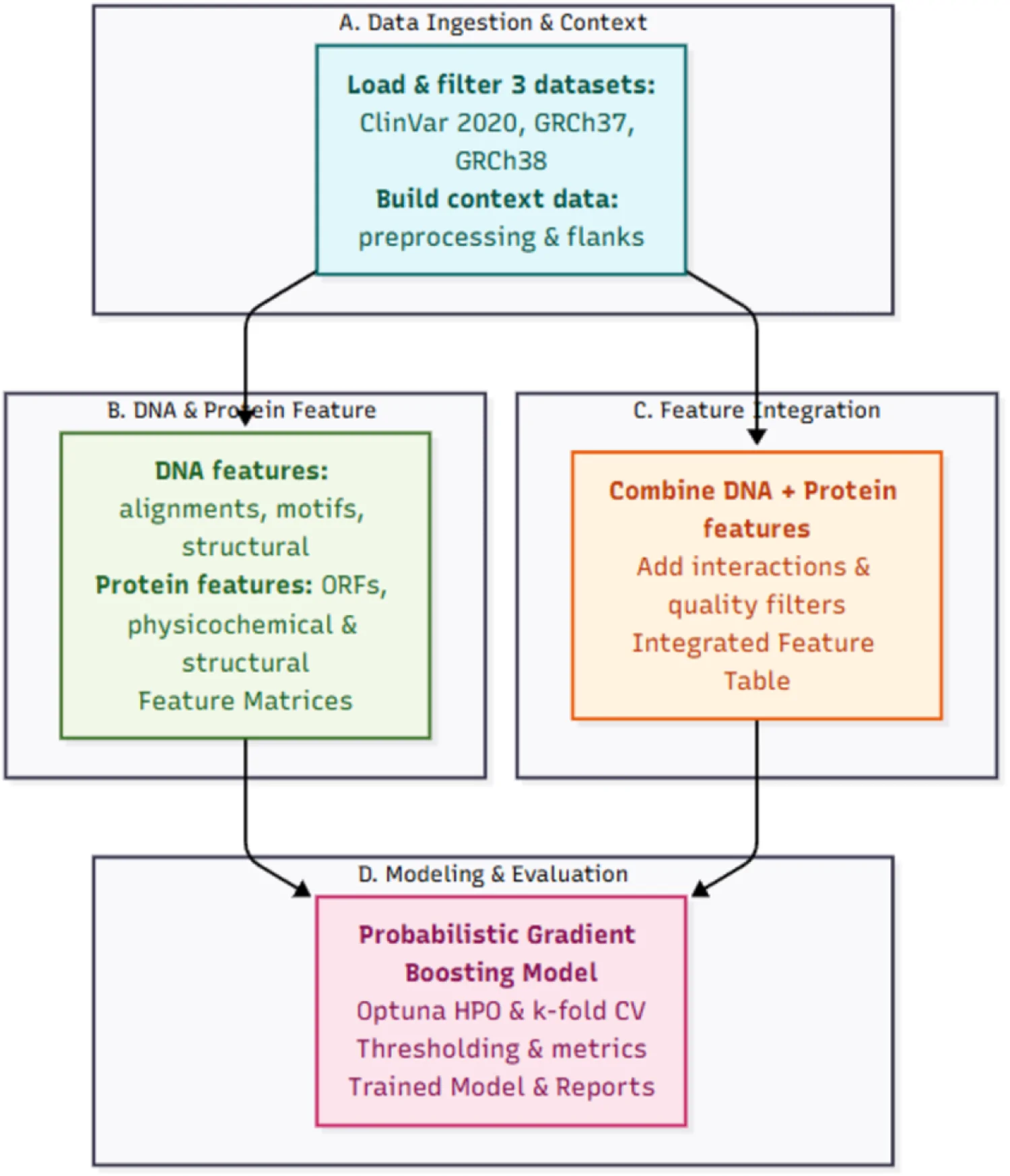
Architecture of machine learning pipeline.

The features extracted are then trained to a probabilistic gradient boosting model [33]. The overall machine learning pipeline architecture is shown in Figure 2. This is a probabilistic method that leaves the old methods of point-estimate boosting strategies and builds its model on the whole predictive distribution. This gives a more formal way to interpret genomic variants, and allows for the joint classification and quantification of uncertainty.

Gradient boosting constructs an ensemble of decision trees by successively adding new learners to the residuals of earlier models. At every step, the algorithm optimizes a differentiable loss using gradient descent in function space. Mathematically, the additive model at iteration *t* is defined as per (Equation 9)

$$F_{t}(x)=F_{t-1}(x)+\eta h_{t}(x)$$ (9)

where $F_{t-1}(x)$ is the model from the previous iteration, $h_{t}(x)$ is the new decision tree fitted to residuals, and *η* is the learning rate controlling the step size.

As mentioned previously, in the case of probabilistic gradient boosting the model does not predict a constant output but rather estimates a probability distribution over outcomes. For binary classification (pathogenic or benign), the predicted probability of the positive class is given by a logistic link function specified in (Equation 10)

$$p(y=1x)=\sigma (F_{T}(x))=\frac{1}{1+e^{-F_{T}(x)}}$$ (10)

where $F_{T}(x)$ is the final ensemble after *T* boosting rounds, and σ(⋅) is the sigmoid function.

The loss function optimized is the negative log-likelihood (cross-entropy), shown in (Equation 11)

$$L=-\frac{1}{N}\overset{N}{\underset{i=1}{\sum }}\;[y_{i}\log\;(\;p_{i})+(1-y_{i})\log\;(1-p_{i})]$$ (11)

where *N* is the number of training samples, yi∈0,1 is the true label, and $p_{i}$ the predicted probability for sample *i*.

To incorporate uncertainty, the probabilistic model extends boosting with variance estimation. Each prediction is modeled as a normal distribution with mean μ(x) and variance $\sigma^{2}(x)$ according to (Equation 12)

$$\hat{y}(x)∼N(\mu (x),\sigma^{2}(x))$$ (12)

where μ(x) is obtained from the boosting ensemble and $\sigma^{2}(x)$ is learned by minimizing a variance-aware objective.

The variance term is optimized through a heteroscedastic likelihood so that the model can represent input-dependent uncertainty. The corresponding objective function is shown in (Equation 13)

$$L_{\mathrm{var}}=\sum_{i=1}^{N}\frac{{\left(y_{i}-\mu (x_{i})\right)}^{2}}{2\sigma^{2}(x_{i})}+\frac{1}{2}\log\;\sigma^{2}(x_{i})$$ (13)

This enables the classifier not only to assign labels but also to quantify confidence in its predictions.

Lastly, the overall model output for a variant *x* is given by the predictive distribution seen in (Equation 14):

$$P(yx)=N(\mu (x),\sigma^{2}(x))$$ (14)

where both the mean and variance are learned from the boosting process.

The probabilistic formulation combines binary classification with uncertainty estimation. The pathogenicity probability is obtained through the logistic transformation of the boosting ensemble output, while a separate variance function estimates input-dependent predictive uncertainty. The logistic component is responsible for class membership prediction, whereas the heteroscedastic variance component quantifies confidence in those predictions. Consequently, the framework produces both a calibrated pathogenicity probability and an associated uncertainty estimate for each variant.

The number of trees, maximum depth of the trees, the learning rate and minimum number of samples per leaf are important hyperparameters of the proposed model. We are using Optuna which is an automated system of hyperparameters optimization [34]. The framework makes good use of the Bayesian optimization and adaptive sampling methods of Optuna to maximize the space of hyperparameters. Once this is done, the model can be fine-tuned for accuracy and generalization purposes. The organized design provides a more structured and directed approach to optimization than random or sub-optimal manual optimization. We now use k-fold cross-validation in order to measure the model quite strongly and prevent overfitting issues. To this the data is divided into k equal folds. To train the model *k* − 1 folds are used and the remaining fold is used to validate the model. This should be repeated k times and the average performance measures are reported.

To ensure optimal model performance and robust generalizability, the probabilistic gradient boosting framework was systematically optimized via automated hyperparameter tuning using the Optuna optimization framework with a Tree-structured Parzen Estimator (TPE) sampler over 100 trials. The hyperparameter search space was explicitly defined as follows: number of estimators $\left(n_{\mathrm{estimators}}\in [500,2500]\right)$, maximum tree depth $\left(\mathrm{ma}x_{\mathrm{depth}}\in [3,12]\right)$, learning rate (η∈[0.01,0.1] log-uniform), subsample ratio $\left(\alpha_{\mathrm{sub}}\in [0.5,1.0]\right)$, colsample by tree ratio $\left(\alpha_{\mathrm{col}}\in [0.5,1.0]\right)$, L1 regularization parameter ($\alpha_{\mathrm{reg}}\in [0.001,10.0]$ log-uniform), L2 regularization parameter (– log-uniform), minimum loss reduction (γ∈[0,5.0]), and the positive class scaling weight $\left(w_{\mathrm{pos}}\in [1.0,5.0]\right)$ to address the intrinsic imbalance in the clinical variant class distributions. Performance was evaluated using a stratified *k*-fold cross-validation strategy (k=5), maintaining consistent pathogenic-to-benign variant ratios across all validation folds. Reproducibility across all data splits and optimization trajectories was enforced by initializing a global random seed of 42. The optimization execution yielded the following optimal hyperparameter configuration: $n_{\mathrm{estimators}}=1,936$, $\mathrm{ma}x_{\mathrm{depth}}=10$, η=0.04198, $\alpha_{\mathrm{sub}}=0.8691$, $\alpha_{\mathrm{col}}=0.9974$, $\alpha_{\mathrm{reg}}=0.2091$, $\lambda_{\mathrm{reg}}=1.6125$, γ=0.3587, and $w_{\mathrm{pos}}=1.8996$.

The main argument for using cross-validation with the k-fold type is that it better reflects the generalization of the models; especially in genomics where the frequency of pathogenic and benign forms of a species might be skewed. The complete algorithm of the proposed pathogenicity prediction framework is shown in Algorithm 1.

**Algorithm 1:** Algorithm of the proposed pathogenicity prediction framework.

function predict_pathogenicity(ClinVar2020, GRCh37, GRCh38): Input: ClinVar2020, GRCh37, GRCh38 Output: R, M Data: Df, F, S, P, *θ*, METRICS Begin // Step 1: Data Preprocessing Df ← LOAD(ClinVar2020, GRCh37, GRCh38) Df ← FILTER(Df, remove_inconsistent ∪ retain_pathogenic ∪ retain_benign) // Step 2: Genome Context Extraction S ← {} for v ∈ Df: S[v] ← EXTRACT_FLANKING_SEQUENCE(v) VALIDATE_ALLELE(v) SAVE(S) // Step 3: Feature Engineering F ← {} for v ∈ Df: fd ← COMPUTE_DNA_FEATURES(v) fp ← COMPUTE_PROTEIN_FEATURES(v) F[v] ← CONCAT(fd, fp) STORE(F) // Step 4: Model Training train_set, test_set ← SPLIT(F) M ← OPTIMIZE_MODEL(train_set, folds = K, method = Optuna) for k ∈ K: TRAIN(M, train_set_k) EVALUATE(M, validation_set_k) SELECT_BEST_MODEL(M) // Step 5: Model Evaluation P ← PREDICT(M, test_set) θ ← ARGMAX_F1(P, test_set) METRICS ← COMPUTE_METRICS(P, θ) PLOT_ROC_PR(P, test_set) SAVE(M) return METRICS, M End

### 3.4 Variant classification module

The Variant Classification Module incorporates the work of the probabilistic gradient boosting model outputs and a decision-making process. Each of the variants is given the probability of being either pathogenic or benign, and a measure of uncertainty. This two-way representation allows the model to trade-off accuracy against interpretability.

The predicted probability of pathogenicity for a given variant x is defined in (Equation 15):

$$P_{\mathrm{path}}(x)=\sigma (F_{T}(x))$$ (15)

where $F_{T}(x)$ is the output of the ensemble after *T* boosting rounds and σ(⋅) denotes the logistic sigmoid function.

A decision boundary is put at a threshold θ, which is used to classify given variants as pathogenic or benign. The classification rule is defined in (Equation 16):

$$\hat{y}(x)=1\;\mathrm{if}\;P_{\mathrm{path}}(x)\geq \theta ,\;0\;\mathrm{otherwise}$$ (16)

where $\hat{y}(x)\in \{0,1\}$ is the predicted class label. 0.5 is the general value for threshold *θ*, but this value can be changed based on clinical requirements such as sensitivity, for specificity trade-offs.

To take into account predictive uncertainty, the variance of the probabilistic gradient boosting model is used to improve classification. The confidence interval of a prediction is presented as follows (Equation 17):

$$CI(x)=\varphi (x)\pm z_{\alpha /2}\cdot \sigma (x)$$ (17)

where φ(x) is the predicted mean probability, σ(x) denotes the standard deviation, and $z_{\alpha /2}$ the critical value from the standard normal distribution. Types that have overlapping confidence measures that are near the threshold can be highlighted in order to be reviewed by hand rather than the automatic classification.

This results in the attainment of the final predictive output by the weighted averaging of the raw probability scores with explicit uncertainty quantification making the classifications both statistically sound and biologically meaningful.

### 3.5 Mutation generation module

The framework contains mutation generation module which examines possible edits to minimise predicted pathogenicity besides classification.

In formal terms, x represents a candidate mutation of sequence x. The optimization objective is given by (Equation 18):

$$M^{*}=\arg\;\underset{M(x)}{\min}\;p(y=1M(x))$$ (18)

*y* = 1 is the pathogenic class. The possible edits of mutation are synonymous mutations or conservative amino acid mutations that maintain functional motifs. Although these are not the outputs meant to be used as clinical recommendations, the module can be used to come up with plausible hypothesis in the experimental study of variant correction.

The mutation generation module identifies sequence modifications that are predicted by the computational model to be associated with lower pathogenicity scores. These results should be interpreted as in-silico hypotheses rather than evidence of biological correction or clinical reversibility. Experimental validation would be required to determine whether any proposed modification produces a genuine functional or therapeutic effect.

## 4 Results and discussion

The section gives the results and experimental design of the proposed probabilistic gradient boosting framework in the variant pathogenicity classification. The results of the proposed model are discussed in terms of benchmark datasets, feature sets, and evaluation metrics and compared to the competitive methods that have already been proposed.

### 4.1 Dataset description

ClinVar 2020 (35), GRCh37 (36), and GRCh38 (37) are those datasets that are deployed in this research. ClinVar is a well-known publicly available database that contains complete data on human genetic variants and their alleged clinical importance that have been gathered by studies, testing labs, and review boards. ClinVar provides variants with an annotation of pathogenic, likely pathogenic, benign, and likely benign, and as supporting evidence of one or the other. The dataset has some major columns that include Variant ID, Chromosome, Position, Reference Allele, Alternate Allele, Clinical Significance, Review Status and Supporting Evidence. These aid in the right mapping and interpretation of the variant effects. GRCh37 and GRCh38 are the reference human genome assemblies, which are currently utilized to align the variants and locate them. Such datasets have different columns, including Chromosome, Start and End Coordinates, Gene Symbol, Transcript ID, Exon Number, and Functional Annotation. We have noted that GRCh38 has better sequence accuracy, gap closures, revised annotations of the genes, additional alternate loci and models of structural variation compared to GRCh37. In this way, more accurate mapping of variants can be achieved, as well as the coverage of intricate genomic regions can be improved. Mapping variants to these reference genomes provides cross-analysis stability, enables linking variants to particular regulatory regions, and enables easy and smooth cross-evaluation across reference genomes. Each of the datasets was purged to eliminate any duplicates and bad records. They were then divided into training, validation, and test sets which were equally represented with classes of the variants.

In this study, the dataset was assembled in various genomic assemblies and clinical repositories to provide a comprehensive evaluation framework. Label noise was reduced by excluding variants from initial training with a “Review Status” of zero stars, as well as those that were “Uncertain Significance”, from the Clinical variant annotations and ground-truth labels were obtained from the ClinVar 2020 release after filtering by strict assertion criteria, retaining only variants with clinical significance “Pathogenic/Likely Pathogenic” (positive class, *y* = 1) or “Benign/Likely Benign” (negative class, *y* = 0). Absolute feature alignment was ensured for baseline models by mapping genomic coordinates and sequence contexts across the GRCh37 and GRCh38 reference assemblies. The final data set was then strictly divided into training, validation and test sets with 70/15/15% data split and stratification to ensure independence of the data sets. This stratification approach allowed the baseline class distributions, genomic context, and specific variant types in each subset to be completely identical, thus preventing data leakage and providing an unbiased assessment of the predictive models.

A total of 520,378 annotated genomic variants (151,931 likely benign, 37,993 likely pathogenic, and 330,454 variants of uncertain significance [VUS]) were used to comprehensively evaluate the framework. Twenty different features were systematically extracted and added to the classification pipeline: SNVs, Microsatellites, Insertion_length, AA_Global_pct, Length_change_context, Stop_codons, Tandem_Duplications, Hairpins, Global_pct, Thermodynamic_stability, TF_motif, Shannon_entropy, Unstable_motif, FI_Mutation_composite, Local_score, FI_General_Stability, Start_codons, GC_context_delta, Local_pct, and Half_life_delta.

GRCh37 and GRCh38 are reference genome assemblies, but they were added to assess the strength of the framework in different genome coordinate systems and different annotations. Mapping variants to both assemblies allows to evaluate the stability of the model with respect to genome annotation, transcript representation and characterization of structural variation. This therefore makes it possible to assess if models are generalizable across reference builds, as consistency of performance across them is evidence of generalizability.

All the analysis and simulation was performed with Python and Python libraries. The data was preprocessed using pandas, the machine learning using scikit-learn and the numerical computations using numpy. Feature extraction, data normalization and model training have been applied as a series of steps. Also the performance of the model was tested with python tool. This consisted of the creation of confusion matrices, ROC curves and the calculation of standard measures of accuracy, precision, recall, and F1-score.

### 4.2 Distribution of variants in ClinVar2020, GRCh37 and GRCh38 datasets

The datasets used in this study consisted of a range of variations characterized by their molecular consequences and span both coding (exonic) and non-coding regions (intronic). They included Intronic variants, which can affect splicing or regulatory elements as well as Missense and Nonsense variants, representing single nucleotide changes in coding regions that may alter or truncate proteins. Frameshift variants resulting from insertions or deletions and UTR variants located in untranslated regions were also present that may have potential impacts on transcript stability and translation. The counts of these variants across the three datasets is shown in Table 1.

**Table 1:** Counts of variants across three datasets.

Dataset	Missense	Intronic	Frameshift	Nonsense	UTR
ClinVar 2020	71,475	37,932	29,387	21,495	11,670
GRCh37	98,975	131,751	79,647	51,854	13,590
GRCh38	98,998	131,762	79,650	51,854	13,590

In ClinVar 2020, the largest category was Missense variants, comprising 71,475 variants (41.03%), followed by Intronic variants with 37,932 variants (21.77%), Frameshift variants with 29,387 variants (16.87%), Nonsense variants with 21,495 variants (12.34%), and UTR variants with 11,670 variants (6.70%). Single nucleotide variants (SNVs), which include Missense and Nonsense variants, therefore constitute the majority of ClinVar 2020 dataset. Minor categories such as in-frame insertions/deletions and other complex changes contributed less than 1% each.

**Figure 3:**
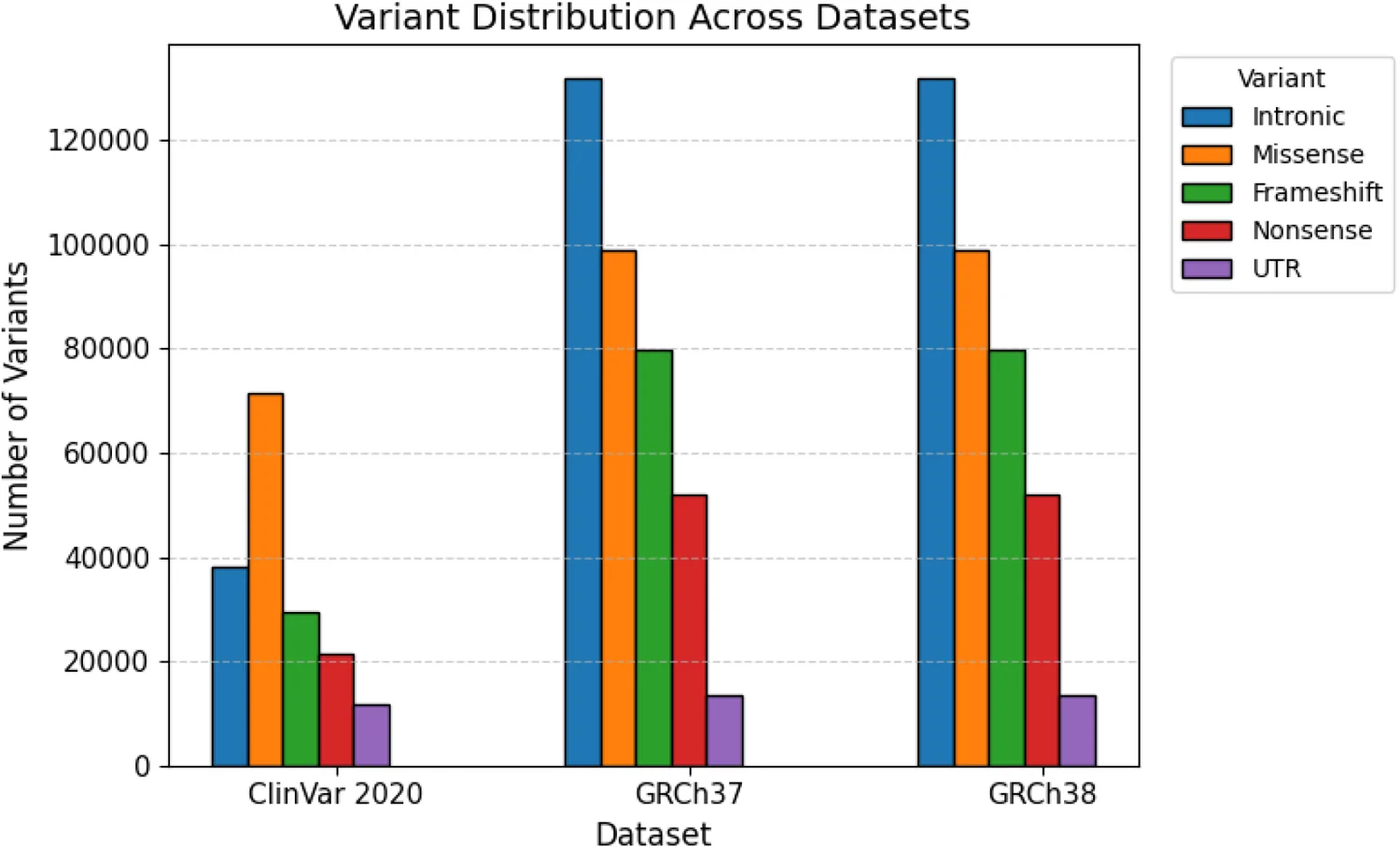
Distribution analysis of variants across datasets.

In GRCh37, Intronic variants represented the largest fraction at 131,751 variants (34.37%), followed by Missense variants with 98,975 (25.82%), Frameshift variants with 79,647 (20.78%), Nonsense variants with 51,854 (13.53%), and UTR variants with 13,590 (3.54%). The distribution in GRCh38 was very similar, with Intronic variants at 131,762 (34.37%), Missense variants at 98,998 (25.82%), Frameshift variants at 79,650 (20.77%), Nonsense variants at 51,854 (13.52%), and UTR variants at 13,590 (3.54%). Figure 3 shows an analysis of the distribution of these variant types across the datasets.

### 4.3 Metrics definition

The metrics that are used to measure the performance of the proposed model in this work are widely accepted modes, i.e., ROC AUC, PR AUC, and F1-Score. A table that is employed to make comparisons between the actual and the predicted class labels is known as the confusion matrix. There are four measures that fall under this category and they are the True Positives (TP), True Negatives (TN), False Positives (FP) and False Negatives (FN). These particular measures were chosen based on the fact that they are the only measures which work well with imbalanced sets of data, like those involved in the pathogenicity classification where the standard accuracy does not offer a meaningful measure of performance.

The Receiver Operating Characteristic Area Under the Curve hereafter called as ROC AUC is used to measure the ability of the classifier to distinguish between classes across different threshold values. The value of ROC AUC is computed by plotting the True Positive Rate (TPR) against the False Positive Rate (FPR) which are in turn derived from the confusion matrix and then calculating the area under the curve. TPR and FPR are calculated as per (Equations 19 and 20). Equation 21 gives the formula for computing the ROC AUC measure.

**True Positive Rate (TPR):** It measures the proportion of actual positives which are correctly identified by the model

$$\mathrm{TPR}=\frac{TP}{TP+FN}$$ (19)

**False Positive Rate (FPR):** It measures the proportion of actual negatives which have been incorrectly identified by the model

$$\mathrm{FPR}=\frac{FP}{FP+TN}$$ (20)

**ROC AUC:** It represents the overall ability of the model to discriminate between classes across different thresholds

$$\mathrm{ROC}\;\mathrm{AUC}=\int_{0}^{1}\mathrm{TPR}(\mathrm{FPR})\;d(\mathrm{FPR})$$ (21)

The Precision-Recall Area Under the Curve (PR AUC) focuses on the relationship between Precision and Recall, making it more informative than ROC AUC when dealing with highly skewed class distributions. It is calculated by plotting Precision against Recall and measuring the area under the curve. Precision and Recall are calculated as per (Equations 22 and 23. Equation 24 gives the formula for computing the PR AUC measure.

**Precision:** It is the measure of proportion of predicted positives that are truly positive

$$\mathrm{Precision}=\frac{TP}{TP+FP}$$ (22)

**Recall:** It represents the proportion of actual positives that have been correctly predicted

$$\mathrm{Recall}=\frac{TP}{TP+FN}$$ (23)

**PR AUC:** It provides a summary of the tradeoff between precision and recall

$$\mathrm{PR}\;\mathrm{AUC}=\int_{0}^{1}\mathrm{Precision}(\mathrm{Recall})\;d(\mathrm{Recall})$$ (24)

The F1-Score is the harmonic means of Precision and Recall, providing a single metric that balances both values. It is useful especially for imbalanced datasets such as the ones used in our study as it penalizes extreme disparities between the Precision and Recall values. The formula for computing F1-Score is given in (Equation 25).

$$F1=2\times \frac{\mathrm{Precision}\times \mathrm{Recall}}{\mathrm{Precision}+\mathrm{Recall}}$$ (25)

In addition to discrimination metrics, we also evaluated the calibration of the probabilistic predictions using the Brier score and Expected Calibration Error (ECE). The Brier score is the mean squared error between predicted probabilities and true binary outcomes, with lower values indicating better calibrated probability estimates. ECE summarizes the discrepancy between predicted probabilities and empirical outcome frequencies across probability bins, thereby quantifying how well nominal risk levels correspond to observed pathogenicity frequencies.

The above metrics together provide a useful way to measure performance of our proposed system. The upcoming section presents and discusses the results of implementing the probabilistic gradient boosting model to the datasets described earlier. Detailed comparisons have also been made with various existing methods.

### 4.4 Performance results

Table 2 represents the performance of the proposed probabilistic gradient boosting model as measured by the ROC AUC, PR AUC, F1-Score, Precision, Recall/TPR, and FPR metrics described above. These metric values have been calculated and tabulated for each of the three datasets ClinVar 2020, GRCh37 and GRCh38.

**Table 2:** Performance of proposed probabilistic gradient boosting model.

Dataset	ROC AUC	PR AUC	F1-Score	Precision	Recall/TPR	FPR
ClinVar 2020	0.9293	0.9361	0.8747	0.8837	0.866	0.1163
GRCh37	0.9610	0.9578	0.9112	0.9139	0.9085	0.0861
GRCh38	0.9646	0.9622	0.9165	0.9157	0.9174	0.0843

**Figure 4:**
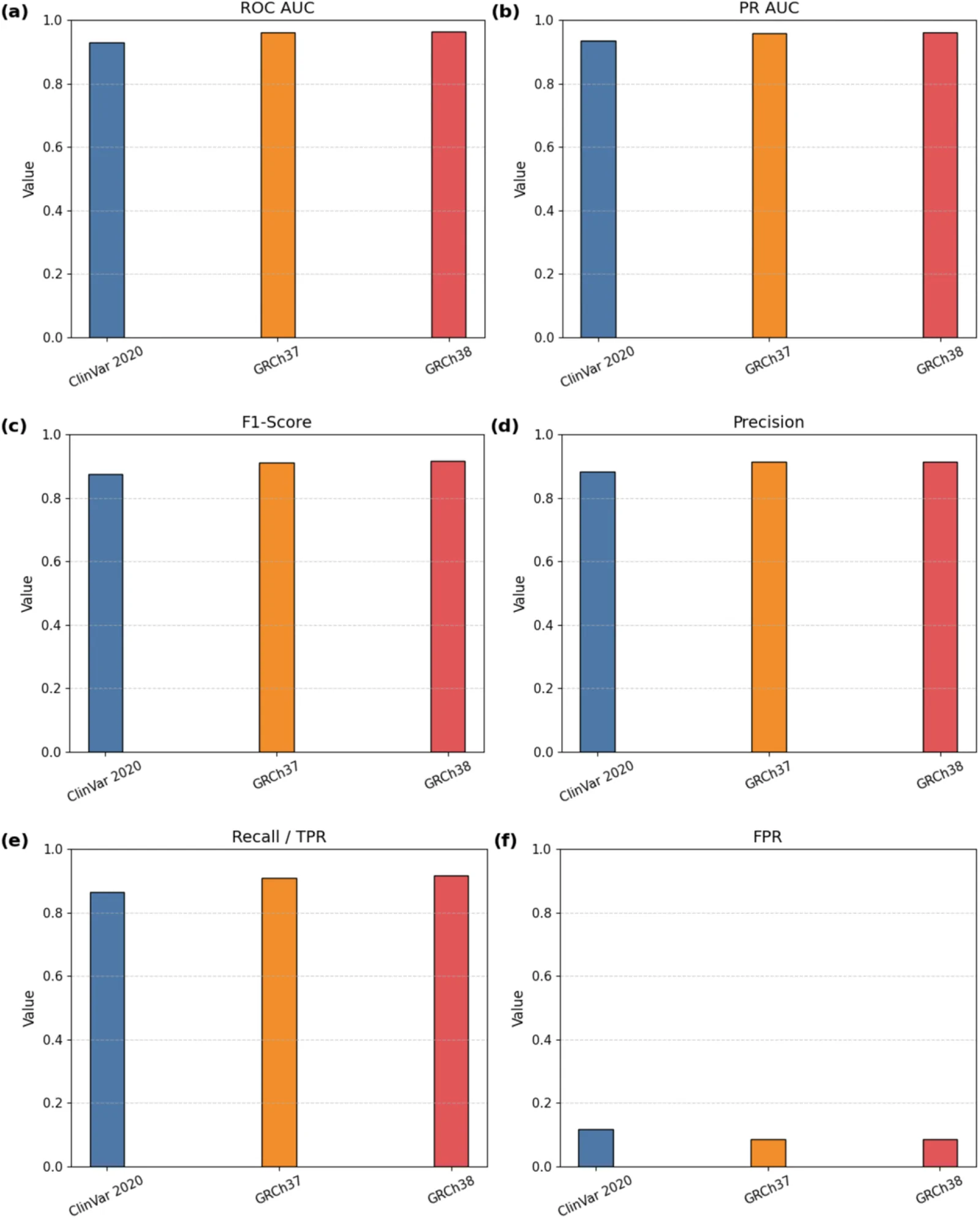
Analysis of proposed model across three datasets (ClinVar 2020, GRCh37, GRCh38) **(a)** ROC AUC, **(b)** PR AUC, **(c)** F1-score, **(d)** precision, **(e)** recall/TPR, **(f)** FPR.

It is observed that the proposed model yields high values particularly when it comes to the ROC AUC and PR AUC measures which are standard benchmarks for evaluating the performance of pathogenicity predictors. The model performs particularly well on the GRCh38 dataset achieving a high ROC AUC of 0.9646 and PR AUC of 0.9622 while it also shows high values for the ClinVar 2020 (PR AUC = 0.9361) and GRCh37 genome dataset (ROC AUC = 0.9610). The proposed model also shows strong Precision values (0.8837, 0.9139, 0.9157) and Recall (or) True Positive Rate values (0.866, 0.9085, 0.9174) across the three datasets respectively while the False Positive Rate is very low (0.1163, 0.0861, 0.0843). The model also performed well in terms of the F1-Score metric, achieving competitive values (0.8747, 0.9112, 0.9165) for all the evaluated datasets. From this we can infer that there is a strong balance between precision and recall especially in identifying the correct pathogenic variant. Figures 4a–f represents the analysis of the probabilistic gradient boosting model across the three datasets with respect to the above metrics.

**Figure 5:**
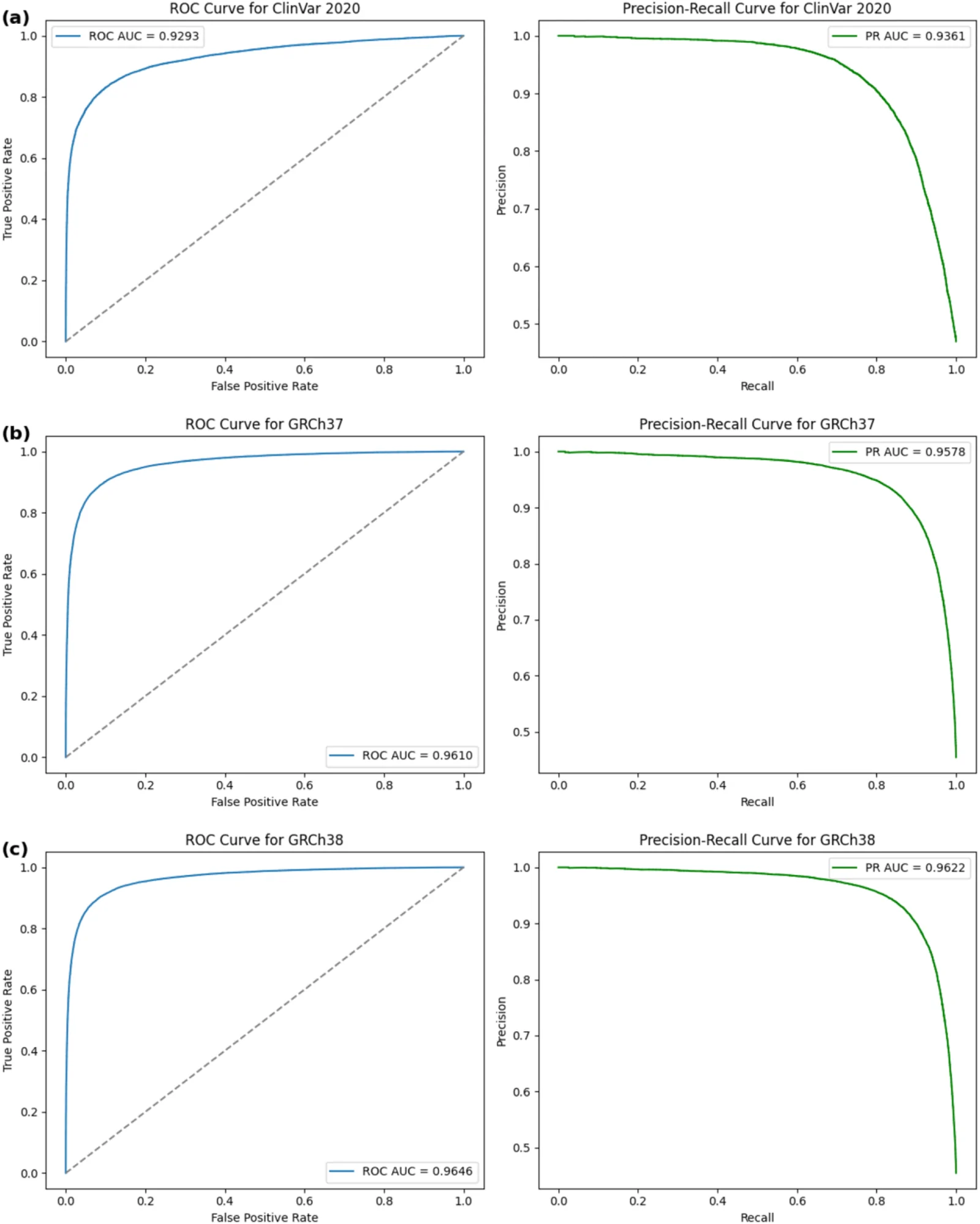
ROC AUC and PR AUC plots for **(a)** clinVar 2020, **(b)** GRCh37, **(c)** GRCh38.

The Receiver Operating Characteristic Area Under the Curve (ROC AUC) and Precision-Recall Area Under the Curve (PR AUC) for each dataset have also been plotted in Figures 5a–c.

**Figure 6:**
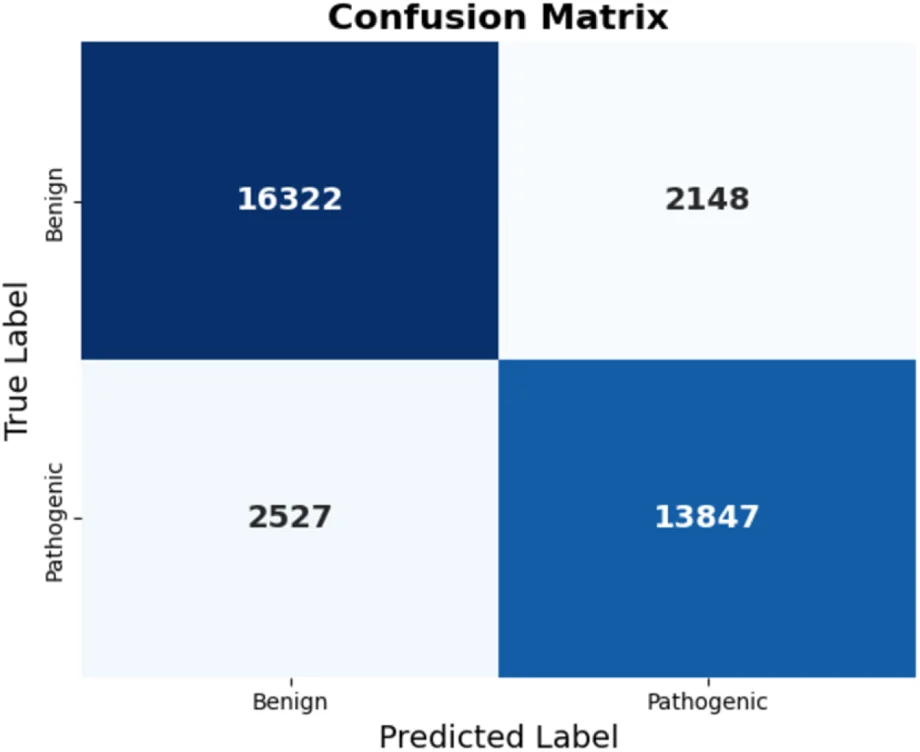
Confusion matrix for ClinVar 2020 dataset.

**Figure 7:**
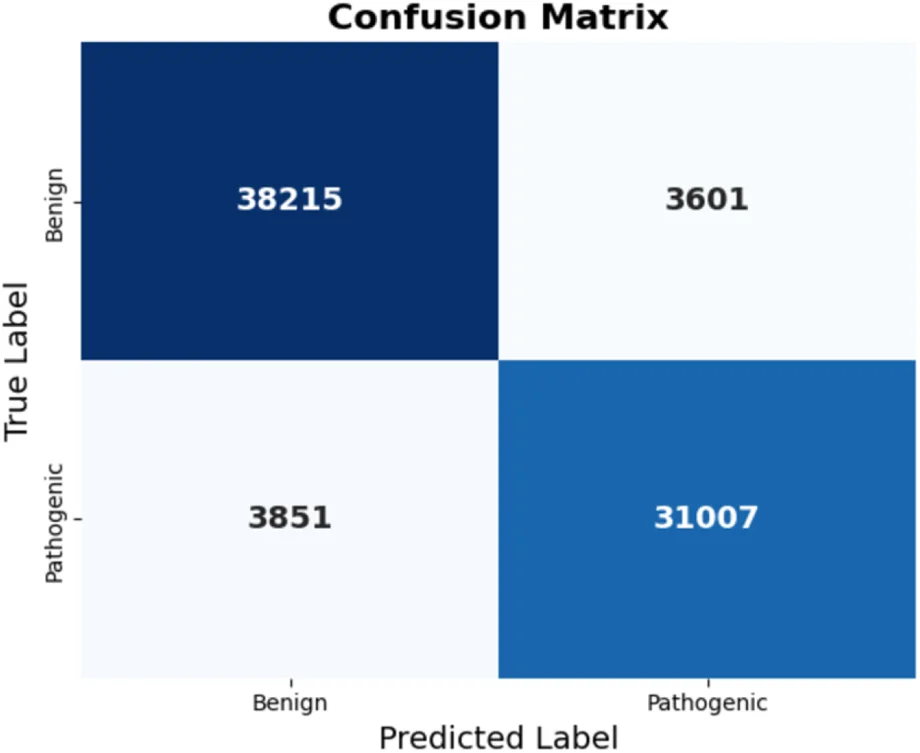
Confusion matrix for GRCh37 dataset.

**Figure 8:**
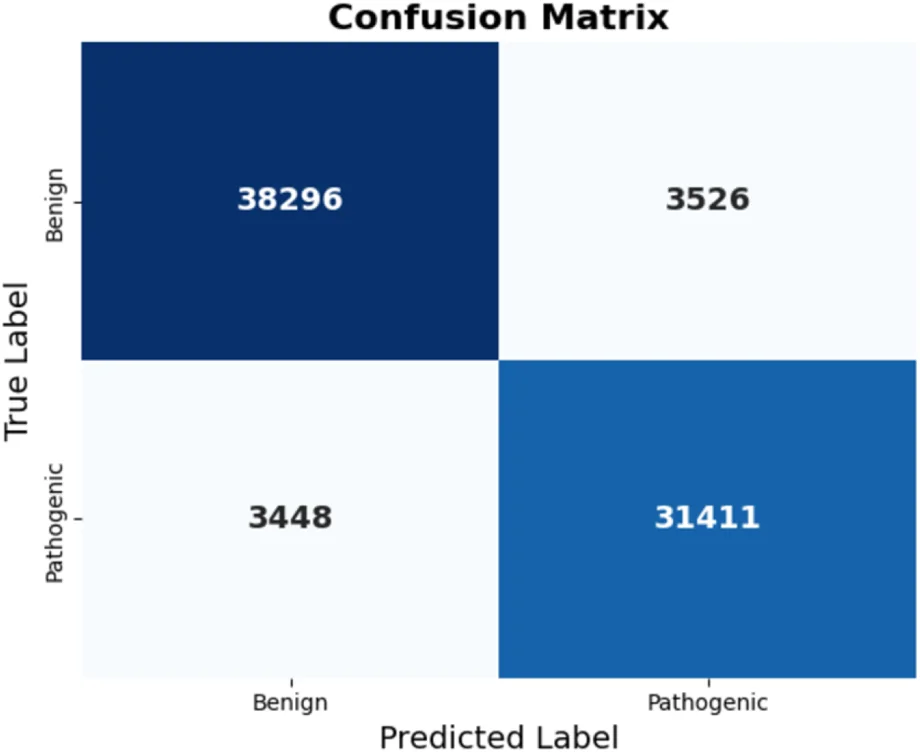
Confusion matrix for GRCh38 dataset.

Confusion matrices have been plotted for all three datasets to visually represent the performance of the proposed model in distinguishing between the pathogenic and benign variants. Across all three, the model shows a high number of correctly classified instances (true positives and true negatives) and relatively few misclassifications (false positives and false negatives) considering the large size of the datasets. Figures 6–8 represents the confusion matrix for ClinVar 2020, GRch37 and GRCh38 datasets respectively.

The proposed model was also used in a mutation generation module to predict pathogenicity and validate the effects whenever a new edit is applied to the input sequence. The input here is in the form of a FASTA consisting of a reference sequence and corresponding alternate allele. It was observed that for one such test FASTA input, the model initially predicted a benignity chance of 3.3% meaning it had a strong pathogenic nature. A series of mutations were then performed on the sequence to try to find out if some possible substitution could be made that could reduce the pathogenicity. During this process multiple variants including *GTC* and *ATC* were identified that showed a very high increase in the benign probability, leading to overall scores crossing 85%. The *ATC* sequence reached the highest benignity probability of 91.5%, which is significantly higher than the initial benignity chance marking an increase of nearly 88%. Therefore, based on the data we can clearly infer that even highly pathogenic variants can be transformed into benign provided suitable mutations are applied. This result does provide some useful insights into the domain of variant correction and interpretation.

The train set was stratified and cross validated into 5 folds to assess the variation between folds. Four subsets of each fold were used to train the model and the remaining subset validated the model while keeping the same class distribution. The model had very low variability across datasets, with standard deviations for ROC-AUC of 0.0020, 0.0011 and 0.0009 for ClinVar 2020, GRCh37 and GRCh38, respectively, and standard deviations for PR-AUC of 0.0028, 0.0012 and 0.0005, respectively. This indicates that the model is not sensitive to the partition of the data, and hence, has good stability across the folds.

**Figure 9:**
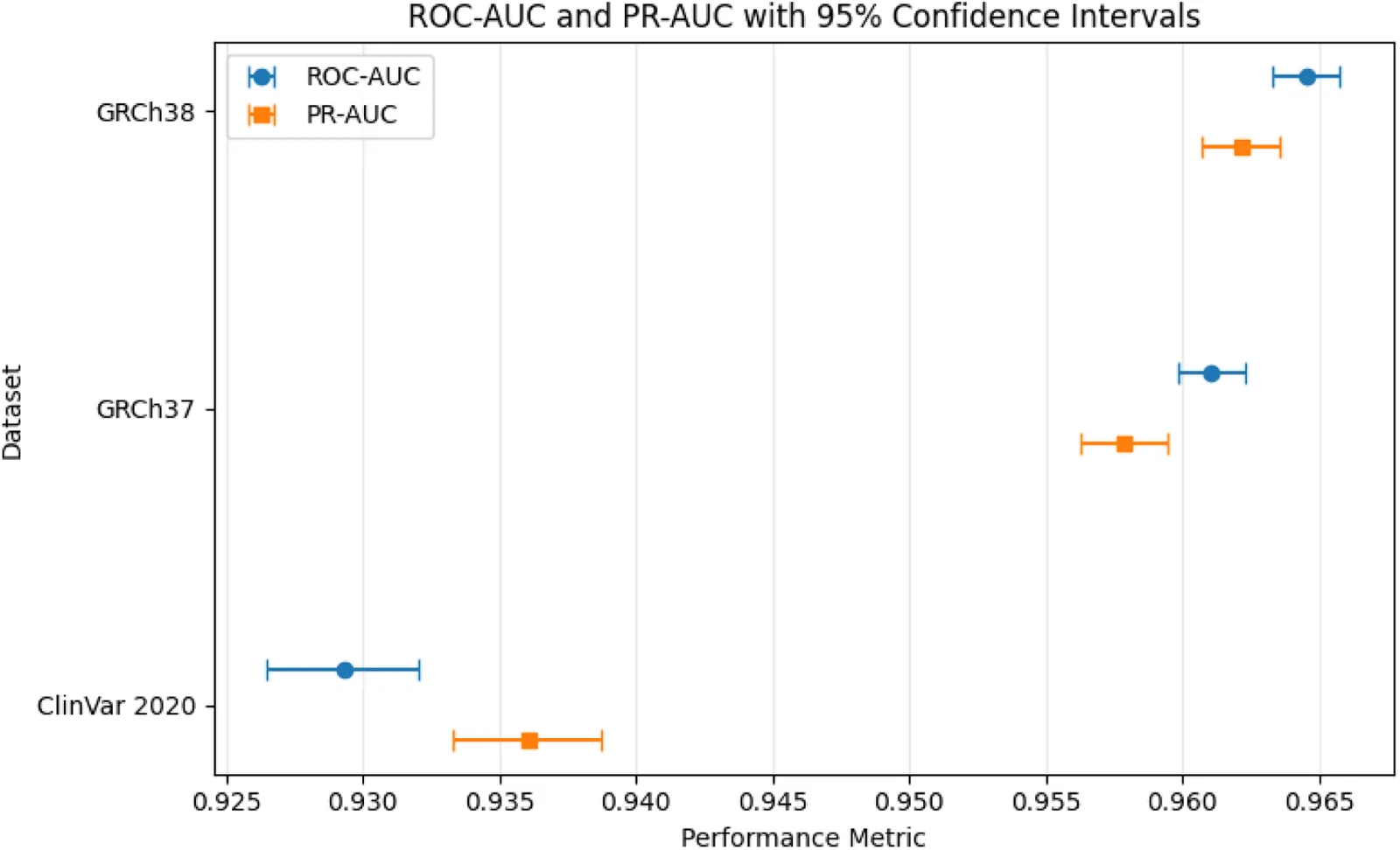
ROC-AUC and PR-AUC confidence intervals across evaluation datasets.

The reported performance was further quantified by running a bootstrap analysis with 1,000 iterations on the held out test sets. The resulting 95% confidence intervals for both ROC-AUC and PR-AUC are shown in Table 3. It was determined that they were consistently narrow for all the datasets. This suggests that the model works well and not much different for various sampling errors. Specifically, the confidence intervals for the ROC-AUC values were close to the point estimates, in the range of about ±0.003. This shows the robustness of the proposed framework on various sets of genomic references. The confidence interval distributions corresponding to them are shown in Figure 9.

**Table 3:** ROC-AUC and PR-AUC with 95% bootstrap confidence intervals across evaluation datasets.

Dataset	ROC-AUC (95% CI)	PR-AUC (95% CI)
ClinVar 2020	0.9293 (0.9265–0.9320)	0.9361 (0.9333–0.9387)
GRCh37	0.9610 (0.9598–0.9623)	0.9578 (0.9563–0.9594)
GRCh38	0.9646 (0.9633–0.9658)	0.9622 (0.9607–0.9636)

### 4.5 Calibration analysis

**Figure 10:**
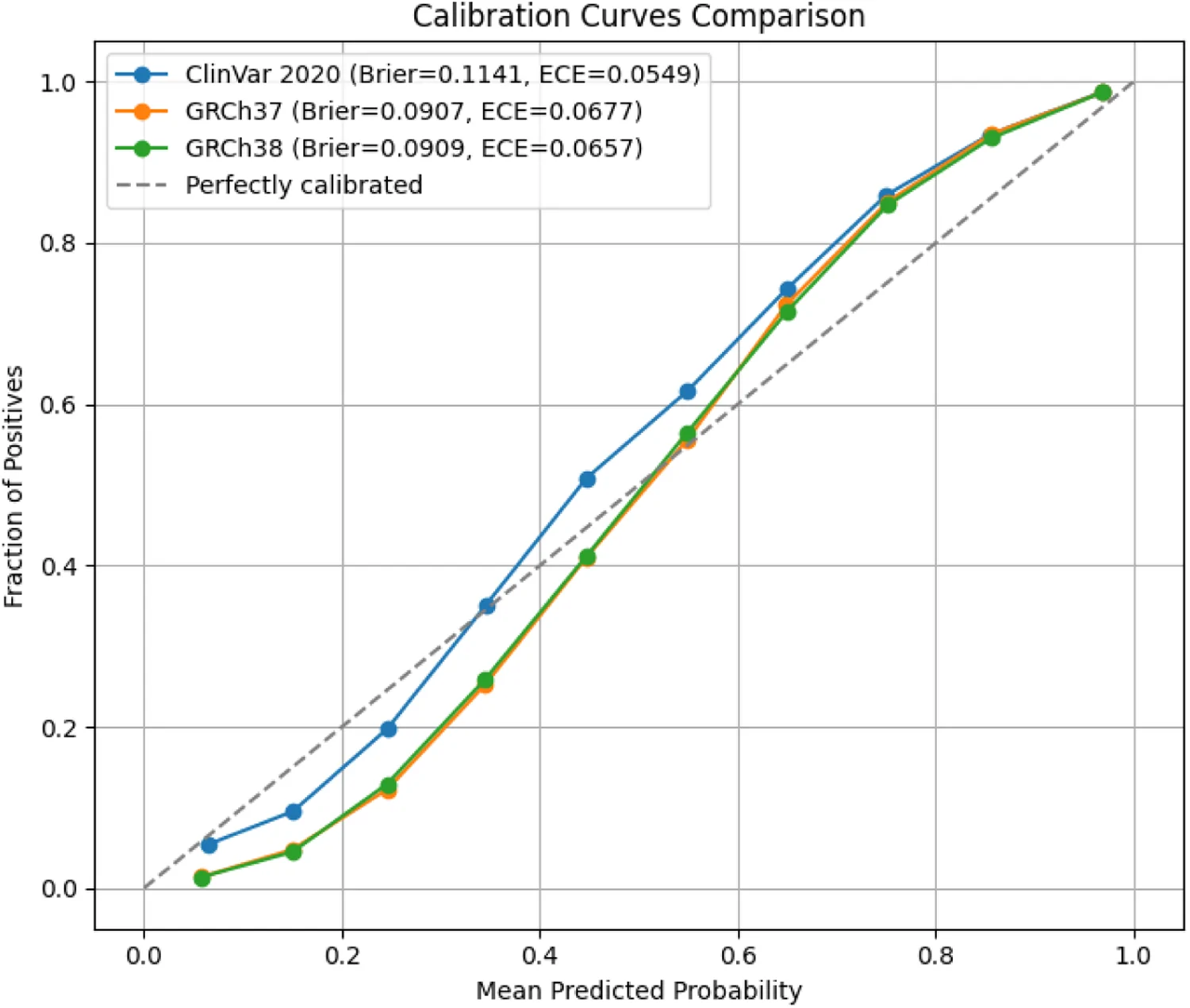
Calibration curve of proposed model across datasets.

The calibration analysis indicates that all three models provide reasonably well calibrated probability estimates. The Brier score of ClinVar 2020 model was 0.1141 with an ECE of 0.0549 and the Brier score of GRCh37 and GRCh38 models was slightly lower with Brier scores of 0.0907 and 0.0909 respectively and ECE was 0.0677 and 0.0657 respectively. These findings indicate that the GRCh37 and GRCh38 models are somewhat more successful in terms of the overall probability estimation but the miscalibration against the true probability model in some probability ranges is very small. But generally speaking the forecasted probabilities are robust in datasets and can be trusted to make interpretations of variants. Figure 10 illustrates the calibration curve plots across datasets.

### 4.6 Comparative analysis

A thorough comparison has also been made between the proposed probabilistic gradient boosting model for pathogenicity classification and several existing methods which have been developed for the same purpose. Table 4 represents this comparison containing the ROC AUC value scores for the proposed method along with a set of recent approaches for the three different datasets. The comparison was made using preceding reference works that have implemented and optimized the models the best on a particular dataset, instead of implementing it locally. This was done to ensure that the models being compared are at their best performance.

**Table 4:** Comparison of proposed method with existing methods on three datasets.

Dataset	Model	ROC AUC
ClinVar 2020	**Proposed**	**0**.**9293**
	EVE (31)	0.911
	DeepSAV (16)	0.894
	FexSplice (19)	0.86
	Optimized Random Forest (18)	0.85
	StrVCTVRE (9)	0.83
	AlphScore (17)	0.793
GRCh37	**Proposed**	**0**.**9610**
	FINSURF (21)	0.957
	VariPred (11)	0.928
	CNVoyant (7)	0.8582
	NeuroCNVscore (8)	0.85
	TADA (12)	0.8865
	PrimateAI (32)	0.797
GRCh38	**Proposed**	**0**.**9646**
	CNVscore (15)	0.945
	VarP (14)	0.95
	AlphaMissense (30)	0.94
	VEST-indel (27)	0.93
	PhenoSV-XGBoost (13)	0.91
	StrVCTVRE (9)	0.83

Bold indicates values corresponding to Proposed Framework in this work.

**Figure 11:**
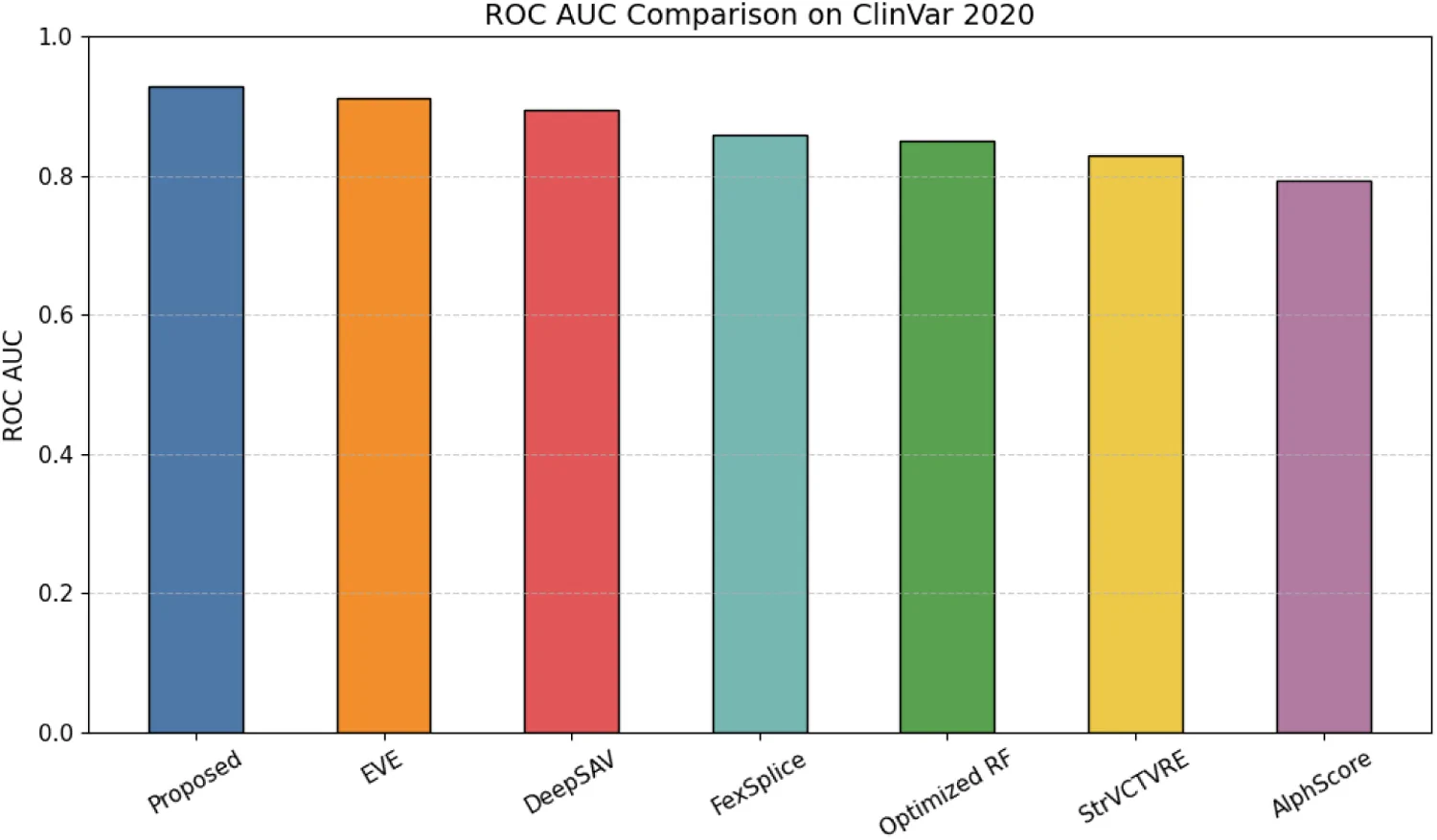
ROC AUC analysis for ClinVar 2020 dataset.

The proposed model demonstrates strong performance across all three datasets. It achieved ROC AUC values of 0.9293 on ClinVar 2020. This is the highest value observed for this dataset (compared to EVE: 0.911, DeepSAV: 0.894, FexSplice: 0.86, Optimized Random Forest: 0.85, StrVCTVRE: 0.83, AlphScore: 0.793). Figure 11 shows the ROC AUC analysis for the ClinVar 2020 dataset.

**Figure 12:**
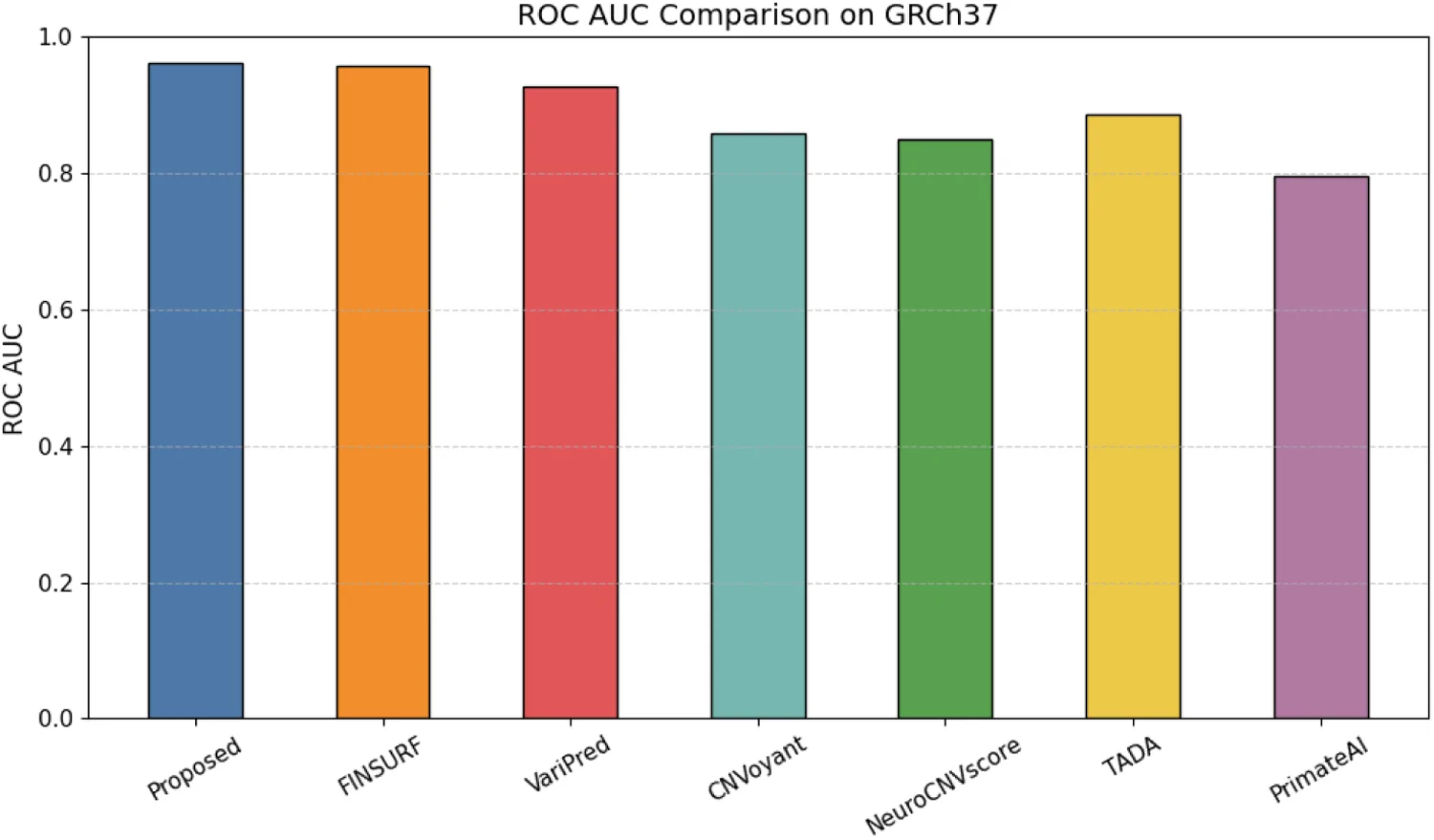
ROC AUC analysis for GRCh37 dataset.

Considering the GRCh37 data, the proposed method achieved 0.9610 ROC AUC. This is again an improvement in value as compared to the existing approaches such as FINSURF: 0.957, VariPred: 0.928, CNVoyant: 0.8582, NeuroCNVscore: 0.85, TADA: 0.8865 and PrimateAI: 0.797. The ROC AUC analysis for GRCh37 is represented in Figure 12.

**Figure 13:**
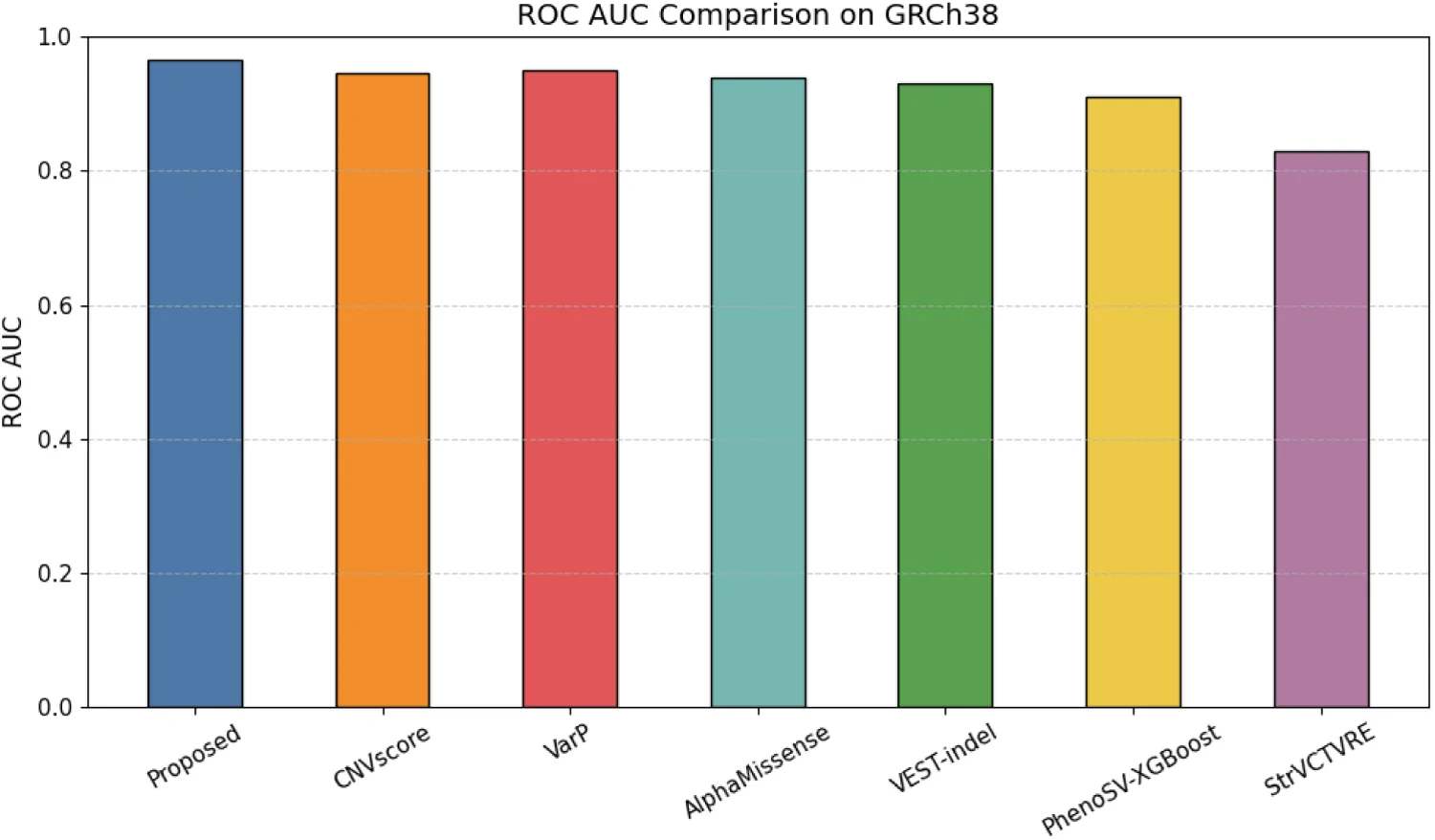
ROC AUC analysis for GRCh38 dataset.

As far as GRCh38 dataset is concerned, the probabilistic model achieves 0.9646 (compared to CNVscore: 0.945, VarP: 0.95, AlphaMissense: 0.94, VEST-indel: 0.93, PhenoSV-XGBoost: 0.91, StrVCTVRE: 0.83). This once again shows how the proposed method outperforms the others. The analysis for GRCh38 is illustrated in Figure 13.

The proposed framework surpasses its existing counterparts in terms of performance, which can be traced to three main reasons. First, it is a model that integrates complementary (CDN) and protein level features, allowing the analysis of sequence, structure, evolutionary and biochemical information at the same time. Second, gradient boosting is able to explain complex non-linear interactions between heterogeneous genomic descriptors and still be interpretable. Third, the probabilistic formulation brings uncertainty quantification via heteroscedastic variance modelling which diminishes overconfident predictions and boosts calibration. This is confirmed by the ablation study, which indicates that the feature representation combination always outperforms the models based on individual feature groups.

Baseline comparisons reported in this study should be considered as indirect comparisons as the reference performance values were derived from previously published studies instead of being re-implemented in a consistent experimental protocol. Reported values may vary due to differences in the composition of the datasets, preprocessing methods and evaluations. The comparison is designed to give contextual benchmarking and not to carry out a head-to-head comparison.

### 4.7 Feature importance analysis

**Figure 14:**
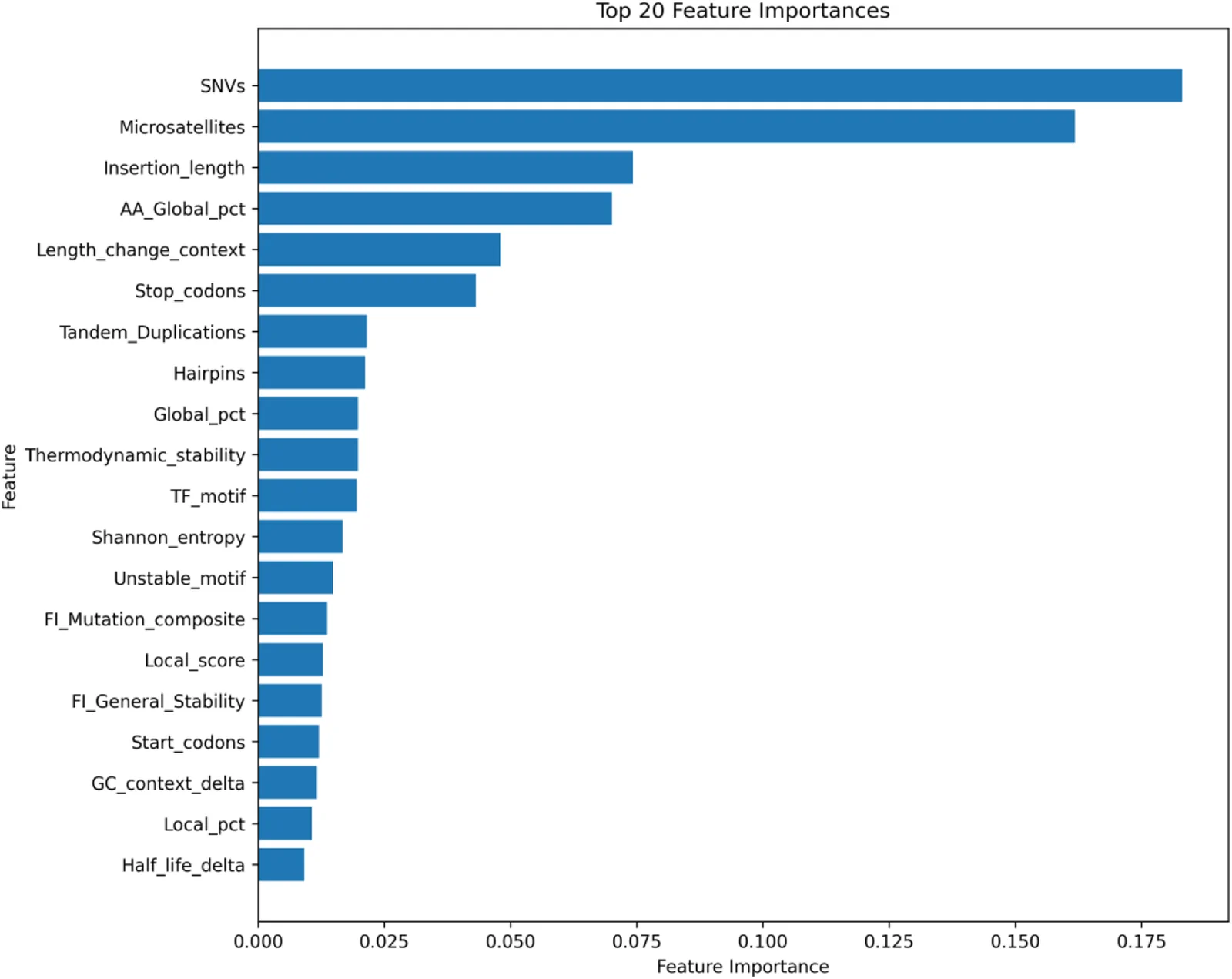
Top 20 most important features ranked by feature importance obtained from the model.

The feature importance analysis provided in Table 5 gives us an idea about the impact the variables have on the model. The most influential features are SNVs (0.1831) and Microsatellites (0.1618) as can be seen in the table. This means that mutation type and patterns of sequence variation are most important in making decisions. The structure of mutations plays a large role, too. Insertionlength (0.0743) and Lengthchangecontext (0.0480) demonstrate the importance of the magnitude of sequence alteration, and context effects. At the protein level consequences, such as the Stopcodons (0.0432) and the Startcodons (0.0121) confirm that the model has been able to model the functional effect of mutations on gene expression and translation. Besides the above, other features that relate to sequence composition and stability such as AAGlobalpct (0.0701) Globalpct (0.0198), Thermodynamicstability (0.0198), and Shannonentropy (0.0168) indicate that nucleotide composition and structural stability are useful in providing valuable contextual information that can be used in prediction. Motif-related and regulatory indicators such as TF_motif, Unstable_motif, and Hairpins also appear among the top-ranked features suggesting that regulatory sequence patterns contribute to the predictive capability of our model. Overall, the results indicate that the model integrates multiple layers of information such as the mutation structure, sequence context, and biochemical or stability properties to effectively evaluate the significance of genomic variations. Figure 14 represents the top 20 most influential features ranked according to their importance scores in the trained model.

**Table 5:** Feature importance scores of the 20 most influential features .

Rank	Feature	Importance
1	SNVs	0.1831
2	Microsatellites	0.1618
3	Insertion_length	0.0743
4	AA_Global_pct	0.0701
5	Length_change_context	0.0480
6	Stop_codons	0.0432
7	Tandem_Duplications	0.0215
8	Hairpins	0.0212
9	Global_pct	0.0198
10	Thermodynamic_stability	0.0198
11	TF_motif	0.0195
12	Shannon_entropy	0.0168
13	Unstable_motif	0.0148
14	FI_Mutation_composite	0.0137
15	Local_score	0.0128
16	FI_General_Stability	0.0126
17	Start_codons	0.0121
18	GC_context_delta	0.0117
19	Local_pct	0.0107
20	Half_life_delta	0.0091

### 4.8 Ablation study

To understand the contribution of different types of features to model performance, we conducted an ablation study. Each feature group was omitted one at a time and the resulting change in performance with respect to the baseline was recorded. The feature groups were identified as genomic_context, sequence_structure, physicochemical, protein_structure, scoring, feature_interactions, and mutation_size. Genomic context features describe the local DNA sequence environment and mutational variability, including regional density, GC content changes, entropy, single nucleotide variants, and insertion lengths. Sequence structure features capture local DNA and RNA motifs, such as tandem duplications, hairpins, microsatellites, codon positions, and transcription factor motifs, which may influence transcription and splicing. Physicochemical features like molecular weight, net charge, isoelectric point, hydrophobicity, aliphatic index, half-life, and proline content capture changes in chemical and physical properties of the protein. The features of the protein structure show the possibility of effect on the secondary structure. Scoring features give composite measures based on other features, whereas feature interactions give non-linear ones. Lastly, mutation size is length of insertions/deletions. Table 6 indicates the values of ROC AUC, PR AUC of each of the feature groups that are omitted and the difference in performance with the baseline model.

**Table 6:** Ablation study results based on different feature groups.

Omitted Feature Group	ROC AUC	Δ ROC AUC	PR AUC	Δ PR AUC
genomic_context	0.8740	0.0553	0.8681	0.0680
sequence_structure	0.8621	0.0672	0.8588	0.0773
physicochemical	0.8778	0.0515	0.8722	0.0639
protein_structure	0.8778	0.0515	0.8730	0.0631
scoring	0.8672	0.0621	0.8628	0.0733
feature_interactions	0.8801	0.0492	0.8746	0.0615
mutation_size	0.8779	0.0514	0.8729	0.0632

**Figure 15:**
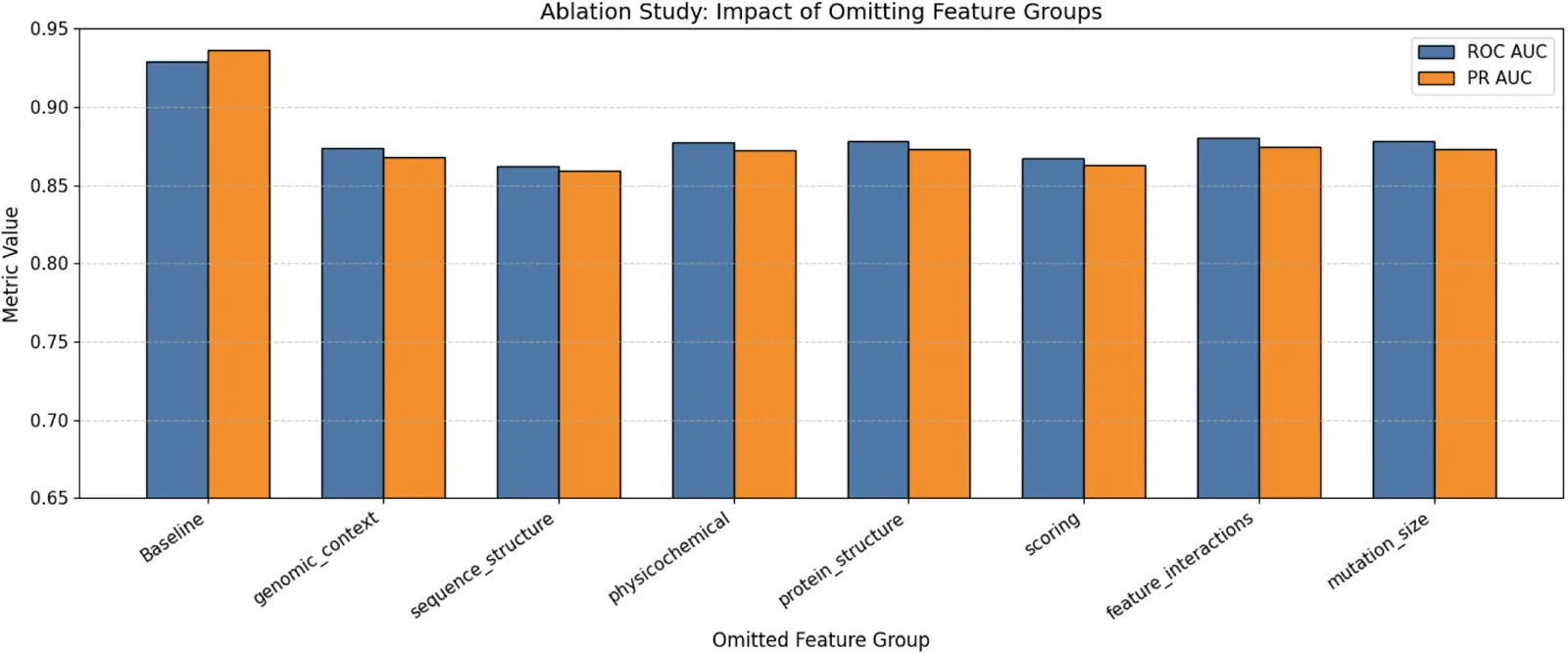
Ablation study results for different feature groups.

Ablation research findings indicate the effect of various collections of features on model performance. The exclusion of sequence structure features resulted in the most significant decrease in ROC AUC (0.067) and PR AUC (0.077) therefore demonstrating the importance of DNA and RNA motifs in variant pathogenicity prediction. Genomic context and scoring features were also significantly affected in terms of omissions that decreased ROC AUC by about −0.051–0.055 and PR AUC by about −0.063–0.068. This means that the sequence attributes of the region and composite measures will be significant in the accuracy of prediction. Feature interactions, which control non-linear relationships between features made a moderate impact, while the removal of physicochemical properties, protein structure, or mutation size were shown to have a smaller impact on the performance reduction. This is indicative that these features are less prominent in conveying information. The baseline model combines all the different groups of features to get the highest performance with the sequence structure and the genomic context as the main determinants. The Figure 15 shows the ablation study results and effect of each feature group in both ROC AUC and PR AUC scores compared to baseline.

### 4.9 Predicting pathogenicity of variants of uncertain significance

To assess the clinical usefulness of the proposed model, it was tested on ClinVar VUS dataset of 520,378 variants of uncertain significance (VUS). The variants included in the dataset were 330,454 variants that were annotated as being of uncertain significance in ClinVar, 151,931 variants that were annotated as likely benign and 37,993 variants that were annotated as likely pathogenic. All the variants of the dataset went through the same feature engineering steps as listed above and the trained model predicted each VUS as pathogenic or benign.

Summary of the predictive outcomes for each ClinVar VUS category presented in Table 7. For variants with benign likelihood annotations, the model had a high rate of agreement: 82.25% of the predicted benigns were annotated as likely benign and only 17.75% as pathogenic. This means that the learned decision boundary is able to reflect characteristics that are consistent with benign variation even when clinical evidence is not definitive. The variants classified as likely pathogenic had a more moderate concordance with 57.82% predicted as pathogenic and 42.18% as benign.

**Table 7:** Results of model predictions for variants of uncertain significance (VUS).

Clinical significance from ClinVar	Variant amount	Number of predicted pathogenic variants (%)	Number of predicted benign variants (%)
Likely benign	151,931	26,974 (17.75%)	124,957 (82.25%)
Likely pathogenic	37,993	21,967 (57.82%)	16,026 (42.18%)
Uncertain significance	330,454	76,073 (23.02%)	254,381 (76.98%)
Total	520,378	125,014 (24.02%)	395,364 (75.98%)

**Figure 16:**
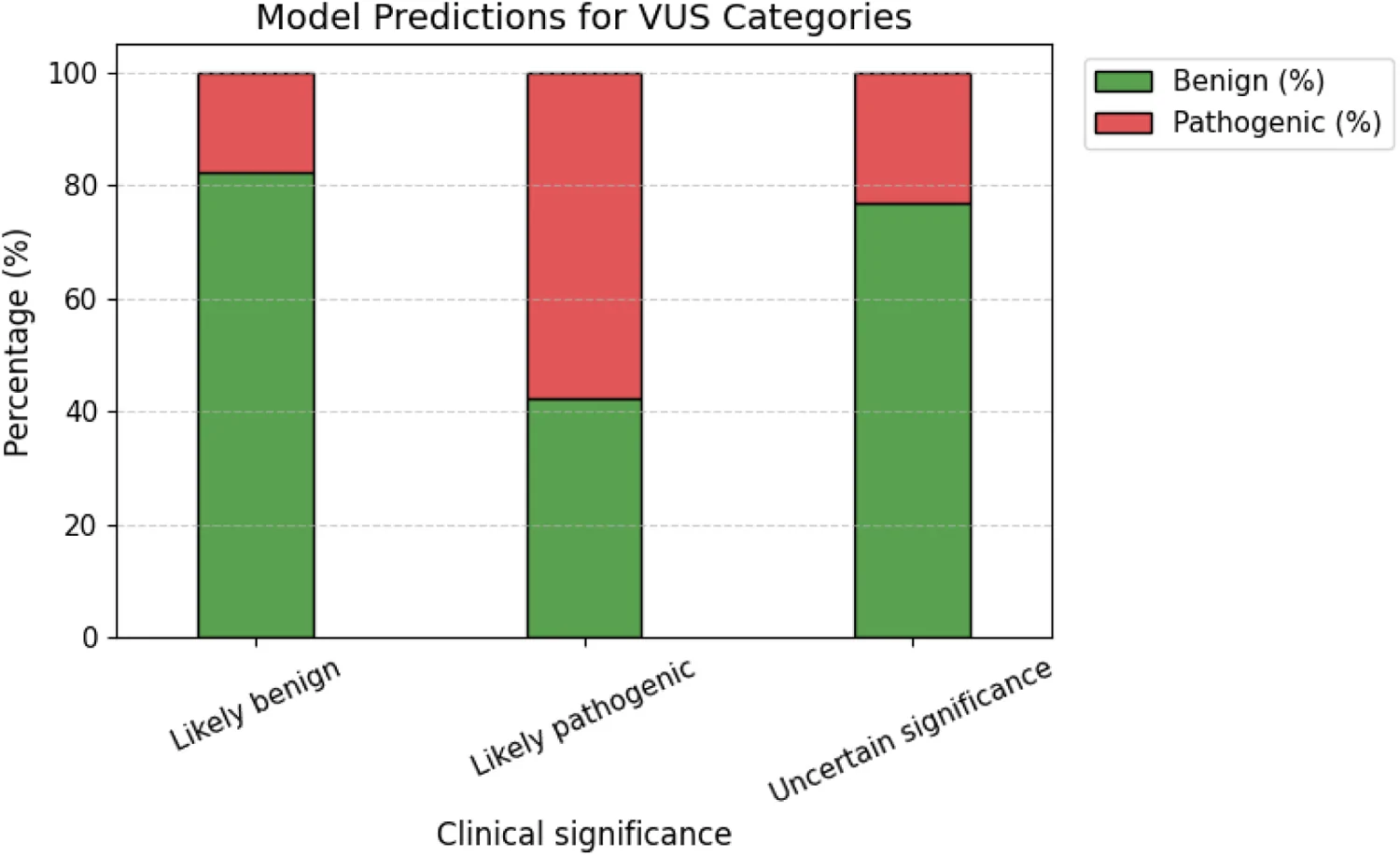
Analysis of model predictions for variants of uncertain significance (VUS).

For variants of uncertain significance which constitute the largest portion of the dataset, the model predicted 23.02% as pathogenic and 76.98% as benign. Although these variants lack clinical validation, this distribution indicates that the model is able to partition VUS into subsets that resemble either benign or pathogenic profiles based on learned patterns. This in turn highlights the potential of the model to provide an additional layer of computational evidence for prioritizing variants for further study. Figure 16 represents the analysis of model predictions for variants of uncertain significance.

## 5 Conclusion

In this study, a machine learning framework using probabilistic gradient boosting model was presented for the purpose of classifying pathogenicity of genetic variants. The proposed method followed an in-depth feature engineering process where a variety of DNA and protein-level features were incorporated prior to the training process. In order to facilitate clinical relevance, the model was trained and tested on widely used benchmark datasets: ClinVar 2020, GRCh37 and GRCh38. The model demonstrated high performance in making predictions and the probabilistic nature contributes to the reliability of these predictions. When compared against multiple available methods, the proposed model was shown to outperform based on metrics such as ROC AUC. The mutation generation module, as previously discussed, provides insights into the potential conversion of pathogenic variants to benign states through targeted mutations. In future iterations, the framework possesses the flexibility to be applied to targeted genomic subsets, such as those specific to diverse population sub-groups. Also, the model could be adapted to rare diseases having limited variant data or Variants of Uncertain Significance (VUS) so that the results can directly support the prevention and cure efforts. The mutation module lacks experimental validation but, in the future, could be integrated into in-silico mutation pipelines used in the laboratory.

## Data Availability

The original contributions presented in the study are included in the article/Supplementary Material, further inquiries can be directed to the corresponding author.
